# Optimization of ultrasound-aided extraction of bioactive ingredients from *Vitis vinifera* seeds using RSM and ANFIS modeling with machine learning algorithm

**DOI:** 10.1038/s41598-023-49839-y

**Published:** 2024-01-12

**Authors:** Selvaraj Kunjiappan, Lokesh Kumar Ramasamy, Suthendran Kannan, Parasuraman Pavadai, Panneerselvam Theivendren, Ponnusamy Palanisamy

**Affiliations:** 1 Department of Biotechnology, Kalasalingam Academy of Research and Education, Krishnankoil, Tamil Nadu 626126 India; 2 School of Computer Science and Engineering, Vellore Institute of Technology, Vellore, Tamil Nadu 632014 India; 3 Department of Information Technology, Kalasalingam Academy of Research and Education, Krishnankoil, Tamil Nadu 626126 India; 4 Department of Pharmaceutical Chemistry, Faculty of Pharmacy, M.S. Ramaiah University of Applied Sciences, Bengaluru, Karnataka 560054 India; 5 Department of Pharmaceutical Chemistry, Swamy Vivekanandha College of Pharmacy, Tiruchengode, Tamilnadu 637205 India; 6 School of Mechanical Engineering, Vellore Institute of Technology, Vellore, Tamil Nadu 632014 India

**Keywords:** Biochemistry, Biological techniques

## Abstract

Plant materials are a rich source of polyphenolic compounds with interesting health-beneficial effects. The present study aimed to determine the optimized condition for maximum extraction of polyphenols from grape seeds through RSM (response surface methodology), ANFIS (adaptive neuro-fuzzy inference system), and machine learning (ML) algorithm models. Effect of five independent variables and their ranges, particle size (*X*_1_: 0.5–1 mm), methanol concentration (*X*_2_: 60–70% in distilled water), ultrasound exposure time (*X*_3_: 18–28 min), temperature (*X*_4_: 35–45 °C), and ultrasound intensity (*X*_5_: 65–75 W cm^−2^) at five levels (− 2, − 1, 0, + 1, and + 2) concerning dependent variables, total phenolic content (y_1_; TPC), total flavonoid content (y_2_; TFC), 2, 2-diphenyl-1-picrylhydrazyl free radicals scavenging (y_3_; %DPPH*sc), 2,2′-azino-bis(3-ethylbenzothiazoline-6-sulfonic acid) free radicals scavenging (y_4_; %ABTS*sc) and Ferric ion reducing antioxidant potential (y_5_; FRAP) were selected. The optimized condition was observed at *X*_1_ = 0.155 mm, *X*_2_ = 65% methanol in water, *X*_3_ = 23 min ultrasound exposure time, *X*_4_ = 40 °C, and *X*_5_ = 70 W cm^−2^ ultrasound intensity. Under this situation, the optimal yields of TPC, TFC, and antioxidant scavenging potential were achieved to be 670.32 mg GAE/g, 451.45 mg RE/g, 81.23% DPPH*sc, 77.39% ABTS*sc and 71.55 μg mol (Fe(II))/g FRAP. This optimal condition yielded equal experimental and expected values. A well-fitted quadratic model was recommended. Furthermore, the validated extraction parameters were optimized and compared using the ANFIS and random forest regressor-ML algorithm. Gas chromatography-mass spectroscopy (GC–MS) and liquid chromatography–mass spectroscopy (LC–MS) analyses were performed to find the existence of the bioactive compounds in the optimized extract.

## Introduction

French Priest introduced grapes in Cumbum Valley, Tamilnadu, India, in 1832, and these grapes were rich in vitamins, tartaric acid, minerals, antioxidants and reduced the risk of some chronic illnesses. Panneer Thratchai (grapes) protect against cancer, heart, nervous diseases and treat haemorrhoids. They prevent Alzheimer’s disease, diabetes mellitus, and protect against oxidative rancidity & viral/fungal infections, also improving night vision^1^. The “Cumbum Panneer Thratchai” grapes (*Vitis vinifera* L., family: Vitaceae; Muscat Hamburg species) grown can be mainly used for the formulation of wine, jam, spirit, canned grape juice, and raisins. During the formulation of grape juice, wine and jam produce large quantities of grape by-products containing seeds, skin, and stalks. Grape’s by-products estimate that the pomace signifies around 20–30% of processed grapes’ weight and 38–52% of seeds^2^. Grape pomace is one of the most common solid by-products generated during the wine-making. Grape pomace is used to recover a wide range of products, including ethanol, tartrates, citric acid, grape seed oil, hydrocolloids, bioactive compounds, and dietary fibre. Grape pomace is one of the significant research areas in the field of fibre extraction, particularly pectin^3^. Importantly, grape seeds are a cost-effective source of antioxidant and potential therapeutic compounds in the form of polyphenols^4^. Grape seeds are described to consist of 11% protein, 35% fibre, 3% minerals, 7% water, 7–20% lipids, and 7% polyphenolic compounds (especially tocopherols and β-carotene)^5^. Polyphenols and other phenolic compounds are gaining attention from scientists because of their potential benefits for human health^6^. Polyphenolic compounds have the property of neutralizing over-generated free radicals (reactive oxygen species (ROS), reactive nitrogen species (RNS), and DNA reactive aldehyde (DRA))^7^. Free radicals are typically generated as a by-product of oxygen metabolism, and mitochondria release it. Free radicals play a dual role; at a low level, they are vital for many cellular signaling mechanisms (i.e., regulate cellular events, like cell cycle, proliferation, migration, and programmed cell death)^8^. In contrast, at a high level, they lead to several pathological complications including damage to protein, nucleic acids, cell, and lipid membrane disturbances, and reduced cellular viability^9^.

Remarkably, an elevated level of ROS causes oxidative stress and the loss of antioxidant and detoxifying enzymes in cells and tissues, as well as oxidative stress itself^9^. An imbalance between a biological system’s capacity to detoxify these reactive chemicals and generating and accumulating reactive oxygen species (ROS) in cells and tissues causes oxidative stress^10^. Numerous studies have demonstrated that oxidative stress and depletion of antioxidant enzymes might have a role in developing and progressing several diseases (such as cancer, diabetes mellitus, cardiovascular diseases (coronary heart disease, atherosclerosis), metabolic disorders, arthritis, and neurodegenerative disorders)^11–14^. Recently, bioactive ingredients from grape seeds gained more attention due to their therapeutic importance^15^. In addition, grape seed powder is a nutraceutical agent usually consumed as a well-being/dietary supplement and sold over-the-counter products in the United States of America^16^. Grape seeds possess numerous polyphenolic compounds, including flavan-3-ols, which act to prevent various diseases^17^. The flavan-3-ols (catechin, epicatechin, epigallocatechin, proanthocyanidin, trans-resveratrol, procyanidin B1, and their polymers) are natural antioxidants (eliminate ROS, RNS, DRA, stimulate detoxifying and antioxidant enzymes) that prevent cell damage and provide other benefits^18,19^. Unfortunately, these bioactive ingredients are present inside the cell in meagre quantities and have a thermolabile structure/character. However, the most sophisticated extraction technique is needed to extract these bioactive compounds from plant sources completely. Compared to modern extraction techniques, traditional methods (such as soxhlation and blending) consume more solvents, take longer time to extraction, and produce less yield of active compounds^20^.

A few advanced extraction techniques, such as microwave-assisted extraction (MAE), pressurized liquid extraction (PLE), ultrasound-aided extraction (UAE), and carbon-dioxide super-critical extraction (CSCE) techniques are followed in the pharmaceutical industries and research laboratories. These advanced extraction techniques are generally called greener and environment-friendly technologies because these processes will consume less energy, permit the use of solvent alternates, renewable natural products, and ensure a safe and high-quality extract/product^21,22^. Carbon-dioxide super-critical extraction is one of the best techniques because it consumes less solvents or eliminates the use of solvents, computer-controllable operations with shorter extraction time, especially thermolabile compounds, are extracted from plant sources without damage^23^. Unfortunately, industries and research laboratories are seeking alternative extraction methods due to the unaffordable cost of carbon-dioxide super-critical extractor^24^. The microwaves from the microwave-assisted extraction (MAE) technique heat the solvent system to enhance the solubility of bioactive compounds of plant cells^25^. While generating heat, it is possible to disintegrate the thermosensitive compounds. This is the major issue for using MAE for thermosensitive compounds^26^. Conversely, ultrasound-aided extraction (UAE) is an exciting and cost-effective alternative for completely extracting plant-derived bioactive ingredients^14^. The UAE method is one of the most preferred extractions, which uses fewer solvents, can be automated at lower temperatures, requires less energy and has a higher yield. It also takes less time to extract the bioactive ingredients. Ultrasonic vibrations accelerate the release of extractable components into the solvent by enhancing mass transport. They also cause rupture of the plant cells by creating physical pressure during ultrasound cavitation^7^.

The present study aimed to maximize the extraction of bioactive ingredients from grape seeds using an ultrasound-aided extraction technique. Many extraction parameters, such as particle size, extraction solvent, solvent concentration, solid-to-liquid ratio, temperature, ultrasonic exposure time, ultrasound intensity, pulse cycle/mode, pH, etc., have potentially influenced the yield of bioactive ingredients and their free radicals scavenging properties^27^. Combining these criteria resulted in the highest yield of bioactive compounds from plant sources, even though they fundamentally appeared unrelated^28^. Combining these parameters must be optimized to achieve the maximum yield of bioactive ingredients^29^. Under this condition, a statistical method of optimization is helpful. One effective method frequently considered for this purpose is the response surface methodology (RSM)^30^. RSM is a statistical technique that determines and simultaneously solves multivariant equations using quantitative data from relevant studies^31^. In RSM, a second-order polynomial equation is applied for modeling and optimization^32^. RSM can be utilized to compare theoretical and actual variables involved in the process with the help of second-order polynomial equations generated in the experiment^33^. Several methods, namely, central composite design (CCD), Box–Behnken design (BBD) and three-level full factorial design (FBD) have been widely applied for RSM to obtain an optimized extraction of bioactive polyphenolic compounds from natural sources^34^. One of the designs used for the application of RSM is CCD, which provides viable models for processes^35^. In contrast to the Box–Behnken design, which is made up of rotated lower-dimensional designs and estimates all linear effects, quadratic effects, and two-way interactions, the CCD is made up of a cube part that is a full factorial that determines main and interaction effects and a star design (α) that quantifies main and quadratic effects^36^. It does not allow for reductions in design, being much less flexible than the CCD. The design space is devoid of any corner points. The factorial portion of the design, which generates rotability, defines the design space box, and the axial points are outside of it. This makes it possible to estimate the expected response with equal variance in any direction with respect to the design space’s centre. Therefore, many researchers have used the central composite design (CCD) to extract bioactive compounds and antioxidants^37^. The (adaptive neuro-fuzzy inference system) and Machine learning algorithm approaches are also used to predict the optimal conditions, producing the best results for nonlinear systems^38^. ANFIS simulates human thought processes using highly developed fuzzy and neural network computer systems^39^. An intelligent neuro-fuzzy method called ANFIS is used to study how variables interact and have nonlinear effects^40^. ANFIS, a hybrid intelligent system^41^. Consequently, multivariable related ambiguous relationships can be quantified using ANFIS through the defuzzification process of the fuzzy inference system (FIS), and error is adjusted for dependable prediction using a backpropagation algorithm with a hidden layer of an artificial neural network (ANN)^42^. Further, a machine learning algorithm adapts the most effective extraction parameters. The aim of machine learning algorithm-based optimization is to reduce the degree of error in a machine learning model, improving its accuracy in making data predictions. Machine learning is generally used to learn the underlying relationship between input and output responses, which is learned from a set of training data^43^. When confronted with new data in a live environment, the model can use this learned approximated function to predict an outcome from this new data. Optimization algorithms can make this process more efficient than any manual process. These algorithms optimize a machine learning model iteratively using mathematical models^44^. The Random Forest Regressor is a simple and widely used algorithm in machine learning^45^. For the best combinations, every hyperparameter configuration is randomly searched and combined. Ultimately, the bioactive ingredients have been recognized using GC–MS (gas chromatography-mass spectrometry) and LC–MS (liquid chromatography-mass spectrometry), indicating the potential of the chosen grape seeds to be used in the healthcare sectors.

## Experimental section

### Materials

#### Grape seeds

Fresh fruits of grapes “Cumbum Panneer Thratchai” (*Vitis vinifera* L., family: Vitaceae; Muscat Hamburg variety) personally collected as a gift sample from grapes farm, Cumbum valley (latitude: 9.734426°, and longitude: 77.280739°), Cumbum (is known as the ‘Grapes city of South India’), Theni District, Tamilnadu, India, on April 2023. The plant material was collected with the consent of the Swamy Vivekanandha College of Pharmacy, Tiruchengode, Tamilnadu, India. No further regulation was required for the collection of this plant. In addition, the collection of plant material complied with the relevant institutional (Swamy Vivekanandha College of Pharmacy), national, and international guidelines and legislation. A pharmacognosist, Professor Murugananthan Gopal M. Pharm., PhD., Principal, Department of Pharmacognosy, Swamy Vivekanandha College of Pharmacy, Tiruchengode, Tamilnadu, India was authenticated grape seeds collected Cumbum (Specimen Number: VV/HER/COG-002). It was deposited at the herbarium, Swamy Vivekanandha College of Pharmacy, Tiruchengode, Tamilnadu, India. The grape seeds were removed from the fruits, and then the separated seeds were washed with tap water and shade-dried for seven days. Thoroughly dried grape seeds were ground into a fine powder using a kitchen mixer grinder (Butterfly Gandhimathi Home Appliances Ltd., Chennai, India). Then, the powdered sample was screened into specified particle size powders (0.15, 0.5, 0.75, 1.0, and 1.35 mm) using an exact sieve (Mesh No. 100, 35, 20, 18, and 14). The powder samples were stored under an airtight container until the start of the experiment (moisture content: 10 ± 2%).

### Chemicals

Ethanol, methanol, chloroform, petroleum ether, ethyl acetate, diethyl ether, acetone, *n*-hexane, gallic acid standard, rutin standard, ascorbic acid, Folin–Ciocalteu’s phenol reagent, sodium carbonate, sodium nitrite, sodium hydroxide, aluminium chloride, ferric chloride, and potassium persulfate were obtained from Himedia Laboratories, Mumbai, India. 2,2′-azino-bis(3-ethylbenzothiazoline-6-sulfonic acid) (ABTS), 2,4,6-tripyridyl-s-triazine (TPTZ), 2, 2-diphenyl-1-picrylhydrazyl (DPPH) were obtained from Sigma Aldrich, Bengaluru, India. All other analytical grade chemicals and reagents were obtained from Himedia Laboratories, Mumbai, India. Distilled water was used for all experiments.

### Ultrasound-aided solvent extractor set-up

The extraction of bioactive ingredients from grape seed powder was performed with an ultrasonic bath extractor (designed by PCI Analytics Ltd., Mumbai, India) having an optimal capacity of 250 mL. The ultrasonic bath has a temperature controller device (accuracy of ± 1.0 °C) with degassing, ultrasound power (220 V), 33 ± 3 kHz operating frequency, continuous mode at 40 kHz high-intensity ultrasound processor, and input voltage range between 200VAC-230VAC, single phase. The ultrasound-instrument produced maximum ultrasound intensity (220 W cm^−2^) with 25 mm titanium probe.

### Methods

#### Preliminary experiments for selection of suitable solvent system

The preliminary experiments were performed to identify the best solvent system for maximum yield of bioactive ingredients based on the highest total phenolic content (TPC), total flavonoid content (TFC), and %DPPH*sc from grape seeds extract. Eight solvents, namely ethanol, methanol, chloroform, petroleum ether, ethyl acetate, diethyl ether, acetone, and *n*-hexane were selected for this investigation. Each solvent system of the extraction process involved using 2 g of grape seed powder (particle size 0.5 mm), 10 mL of fixed solvent concentration (70% V/V in distilled water), ultrasound intensity: 60 W cm^−2^, ultrasound exposure time: 10 min, and temperature: 40 °C. Using a UV–Visible spectrophotometer, Shimadzu UV-1800 series, and UV Probe 2.62 software, Japan, to measure the concentration of bioactive ingredients from grape seed extract. A rotary vacuum dryer (Buchi rotary evaporator, Mumbai, India) was used to concentrate the extracts. The concentrated extracts were then lyophilized (freeze dryer) to convert into powder form and stored in a desiccator until the experiment.

#### Selection of relevant extraction variables

Five independent extraction variables were selected, i.e., particle size (*X*_1_; in mm), solvent concentration (*X*_2_; in % V/V with distilled water), ultrasound exposure time (*X*_3_; in min), temperature (*X*_4_; in °C), and ultrasound intensity (*X*_5_; in W cm^−2^), basis on the dependent variables, such as total phenolic content (TPC; y_1_), total flavonoid content (TFC; y_2_), and their antioxidant potentials (DPPH free radical scavenging (y_3_), ABTS free radical scavenging (y_4_), and FRAP potential (y_5_)). According to the preliminary experimental findings, methanol is an ideal solvent for extracting grape seeds’ highest concentration of beneficial compounds, as shown in Table 1. The selected five independent variables were investigated at five-coded levels, such as very low (− 2), low (− 1), medium (0), high (+ 1), and very high (+ 2), and each variable range presented in Supplementary Table 1. The selected independent variable ranges were particle size (*X*_1_: 0.5–1 mm), methanol concentration (*X*_2_: 60–70% in distilled water), ultrasound exposure time (*X*_3_: 18–28 min), temperature (*X*_4_: 35–45 °C), and ultrasound intensity (*X*_5_: 65–75 W cm^−2^) investigated.

**Table 1:** Preliminary selection of appropriate extraction solvent.

Solvents	TPC* in mg GAE/g	TFC* in mg RE/g	%DPPHsc*
Ethanol	543.36 ± 2.57	348.64 ± 2.5	65.34 ± 1.04
Methanol	548.52 ± 7.15	354.28 ± 2.85	67.28 ± 1.95
Chloroform	474.72 ± 4.05	301.43 ± 1.04	53.34 ± 1.17
Petroleum ether	523.56 ± 1.19	335.72 ± 2.16	58.43 ± 0.76
Ethyl acetate	518.34 ± 1.77	321.26 ± 3.68	55.76 ± 0.51
Diethyl ether	528.35 ± 2.65	338.39 ± 1.95	59.53 ± 1.14
Acetone	504.2 ± 2.04	307.32 ± 0.72	52.38 ± 1.07
*n*-Hexane	472.23 ± 2.08	303.85 ± 1.17	51.56 ± 1.06

*All the experiments were repeated three times and values are expressed as mean ± standard deviation.

#### Procedure for ultrasound-aided extraction (UAE) of bioactive ingredients

The extraction of bioactive ingredients from grape seeds powder was performed using an adjustable ultrasonic bath extractor with a sample of 2 g grape seeds powder in a closed container, which contained 10 mL solvent (miscible methanol in water), and specified particle size, ultrasound exposure time, temperature and ultrasound intensity. According to the Response Surface Methodology’s (RSM) central composite design (CCD), experiments were conducted in triplicate. After UAE, the extracts were filtered using Whatman No. 1 filter paper and filtrate was centrifuged at 3500 rpm for 30 min at 4 °C. The supernatant liquid (extract) was concentrated at 40 °C using a rotary vacuum dryer. The concentrated methanolic extract was then lyophilized (freeze dryer) to convert it into powder form to determine the TPC, TFC, and antioxidant potentials (%DPPH*sc, %ABTS*sc, and FRAP).

#### Determination of total phenolic content (TPC)

The spectrophotometric analysis determined the quantity of TPC (y_1_) present in the extract according to the previously described method^46^. Briefly, 0.2 mL of grape seeds extract was mixed individually with 5 mL of 10% resolubilized Folin–Ciocalteu reagent. 2 min vortex the mixture, and 2 mL of 7.5% sodium carbonate (Na_2_CO_3_) was added to the mixture after 5 min. The samples were kept in the dark at room temperature for an hour. They used a UV–Visible spectrophotometer, Shimadzu UV-1800 series, and UV Probe 2.62 software, Japan, to measure the absorbance at 765 nm. The results were presented as mg of gallic acid equivalent (GAE) per gram of sample using gallic acid as the reference standard.

#### Determination of total flavonoid content (TFC)

The TFC (y_2_) was determined by the method developed by da Silva et al.^47^. 1 mL of resolubilized extract sample mixed with 0.3 mL of 5% sodium nitrite. The mixture was allowed to incubate for 6 min in a dark place, and then 0.3 mL of 10% aluminium chloride solution was added. 3 mL of 1 M sodium hydroxide was added to the reaction, and the incubation was continued for 10 min. After 10 min, a UV–Visible spectrophotometer was employed to measure the absorbance at 510 nm. The results were presented as mg of rutin equivalent (RE) per gram of sample.

### Determination of antioxidant potential

#### %DPPH scavenging assay

The DPPH free radical scavenging potential (y_3_) of grape seed extracts was determined according to Musa et al.^48^. Concisely, 3 mL of DPPH free radical solution (0.1 mM DPPH in ethanol) was mixed with 0.1 mL of grapes seeds extract; the mixture was then incubated at 37 °C for 30 min. After incubation, a UV–Visible spectrophotometer was used to quantify the absorbance at 517 nm. Methanol and DPPH were both employed as controls. The % DPPH radical scavenging ability was calculated as Eq. (1):

1 $$\% {\text{ DPPH radical scavenging activity }} = \, \left( {\left( {{\text{A}}_{0} - {\text{A}}_{{1}} } \right) \, \times {1}00} \right)/{\text{A}}_{0} ,$$

where A_0_—absorbance of the control and A_1_—absorbance of the sample.

#### %ABTS scavenging assay

The ABTS free radical scavenging potential (y_4_) of grape seed extract was determined according to Canabady-Rochelle et al.^49^. Concisely, 2.45 mM of potassium persulfate was mixed with 7 mM ABTS radical solution, and the resultant reaction mixture was stored in the dark for 16 h at room temperature. Then, ethanol was used to adjust the reaction mixture’s absorbance to 0.70 ± 0.05 at 734 nm. 1 mL of this reaction mixture was mixed with 10 µL of grape seed extract. The absorbance was measured against the blank reagent at 734 nm. The inhibition activity was determined by the following Eq. (2):

2 $$\% {\text{ ABTS radical inhibition activity }} = \, \left( {\left( {{\text{A}}0 - {\text{A1}}} \right) \, \times {1}00} \right)/{\text{A}}0,$$

where A0—absorbance of the control and A1—absorbance of the sample.

#### The ferric-reducing antioxidant potential (FRAP) assay

The FRAP (y5) of grapes seed extract was carried out based on the FRAP technique^50^ and modified by Pulido et al.^51^. The FRAP reagent was prepared using 300 mM acetate buffer (3.1 g sodium acetate in 16 mL acetic acid at pH 3.6), 10 mmol TPTZ, and 20 mmol FeCl_3_·6H_2_O and 4mMol hydrochloric acid in the ratio of 10:1:1. 0.1 mL of grapes seed extract was mixed with 3.0 mL FRAP reagent and incubated in darkness for 30 min at 37 °C. The absorbance was read at 595 nm using a UV–Vis Spectrophotometer. The standard curve was linear through 200 and 1000 μM FeSO_4_. Results calculated in μM Fe (II)/g dry mass were compared with ascorbic acid as a standard.

### Experimental design and optimization using RSM

A five-level, five-coded variable central composite design (CCD) in Response surface methodology (RSM) was applied to optimize the effective extraction parameters of ultrasound-aided extraction technique concerning TPC, TFC, and antioxidant potentials (DPPH*sc, ABTS*sc, and FRAP) from grape seed extract. The selected five-coded variables were particle size (*X*_1_: 0.5–1 mm), methanol concentration (*X*_2_: 60–70% in distilled water), ultrasound exposure time (*X*_3_: 18–28 min), temperature (*X*_4_: 35–45 °C), and ultrasound intensity (*X*_5_: 65–75 W cm^−2^) at five levels deficient (− 2), low (− 1), medium (0), high (+ 1), and very high (+ 2) investigated for maximum yield of bioactive ingredients from grape seeds extract. The independent variables were coded based on the below Eq. (3):

3 $$x_{i} = \frac{{X_{i} - X_{Z} }}{{\Delta X_{i} }}\quad i = 1,2,3,...K,$$

where *xi* is the dimensionless value of the independent parameter; *X*i, is the actual value of an independent parameter; *Xz*, is the actual value of an independent parameter at the central point; and Δ*Xi*, is the step change of the actual value of the parameter *i* representing to a variation of a unit for the dimensionless value of the parameter *i.* The total number of experiments was calculated from Eq. (4), which is given below:

4 $${\text{N }} = { 2}^{{\text{k}}} \left( {\text{factorial points}} \right) \, + {\text{ 2k }}\left( {\text{axial points}} \right) \, + {\text{ n}}_{0} \left( {\text{central points}} \right),$$

where N is the total number of experiments, k is the independent variable number, and n_0_ is the replicate number at the central points, resulting in an experimental design of 50 runs. Fitting experimental data determined the correlation between the dependent and independent variables in a second-order polynomial regression model. In the case of these 50 experimental runs comprised of 32 factorial points, 8 repeated levels of central points, and 10 axial points (α) at a distance of ± 2 from centre points is shown in Table 2. The results of the CCD studies (Table 2) were analysed employing the multiple regression equation.

5 $$Y = \alpha_{0} + \sum\limits_{i = 1}^{3} {\alpha_{i} X_{i} } + \sum\limits_{i = 1}^{3} {\alpha_{ii} X_{i}^{2} } + \sum\limits_{i = 1}^{2} {\sum\limits_{j = i + 1}^{3} {\alpha_{ij} X_{i} X_{j} } } + \varepsilon .$$

**Table 2:** Central composite design (CCD) with experimental responses and predicted responses.

S.No	Parameters					Experimental value*					RSM prediction					ANFIS prediction					Machine learning algorithm prediction				
	*X*_1_	*X*_2_	*X*_3_	*X*_4_	*X*_5_	y_1_	y_2_	y_3_	y_4_	y_5_	y_1_	y_2_	y_3_	y_4_	y_5_	y_1_	y_2_	y_3_	y_4_	y_5_	y_1_	y_2_	y_3_	y_4_	y_5_
1	1	60	18	45	65	432.26	312.34	62.65	59.45	54.67	432.03	298.47	62.04	58.87	53.90	432	312	62.7	59.5	54.7	643.54	411.64	76.84	71.12	66.30
2	1	70	18	45	75	462.56	325.58	65.43	61.92	56.58	447.98	309.29	62.20	58.14	53.02	463	326	65.4	61.9	56.6	572.68	346.90	66.05	62.82	57.70
3	0.5	60	18	35	75	599.06	391.56	72.34	66.45	61.06	600.04	382.65	73.64	67.52	62.77	599	392	72.3	66.4	61.1	643.54	411.64	76.84	71.12	66.30
4	0.155	65	23	40	70	670.32	451.45	81.23	77.39	71.55	739.73	466.89	83.08	77.92	72.81	670	451	81.2	77.4	71.6	643.54	411.64	76.84	71.12	66.30
5	1	70	28	45	75	562.56	353.03	68.03	65.4	60.48	511.45	336.76	66.65	62.19	57.47	563	353	68	65.4	60.5	631.10	393.11	74.63	68.02	63.66
6	0.5	70	18	35	65	653.28	403.06	76.27	72.04	68.76	616.82	393.6	75.529	71.296	66.94	653	403	76.3	72	68.8	643.54	411.64	76.84	71.12	66.30
7	0.75	65	23	28.11	70	498.34	334.56	67.83	63.45	57.46	495.19	328.22	64.787	59.87	54.62	498	335	67.8	63.5	57.5	631.10	393.11	74.63	68.02	63.66
8	1	70	18	45	65	427.56	319.48	65.84	61.5	57.36	449.98	314.75	64.64	61.48	57.24	428	319	65.8	61.3	57.4	643.54	411.64	76.84	71.12	66.30
9	1	60	18	35	65	431.67	320.25	65.82	62.87	58.26	432.81	291.18	61.55	56.50	52.15	432	320	65.8	62.9	58.3	417.93	301.03	61.55	57.98	53.38
10	0.75	76.89	23	40	70	513.63	367.58	66.61	63.65	59.34	552.59	364.22	66. 4	63.330	59.21	514	368	66.6	63.7	59.3	631.10	393.11	74.63	68.02	63.66
11	1	60	28	45	75	423.78	321.25	61.64	57.72	52.08	458.15	311.1	60.17	55.92	51.12	424	321	61.6	57.7	52.1	572.68	346.90	66.05	62.82	57.70
12	0.5	70	18	45	75	659.43	413.76	77.59	73.04	68.56	629.26	401.48	78.11	73.21	68.54	659	414	77.6	73	68.6	643.54	411.64	76.84	71.12	66.30
13	0.5	60	18	45	65	642.57	396.56	76.06	71.43	65.33	647.3	399.68	73.18	69.23	63.91	643	397	76.1	71.4	65.3	417.93	301.03	61.55	57.98	53.38
14	0.5	60	28	45	65	660.12	422.6	63.45	59.93	55.52	629.26	401.58	66.50	62.43	57.43	660	423	63.5	59.9	55.6	631.10	393.11	74.63	68.02	63.66
15	1	70	18	35	75	414.56	299.39	52.02	48.93	43.78	408.63	284.88	55.78	51.62	47.28	415	299	52	48.9	43.8	417.93	301.03	61.55	57.98	53.38
16	1	60	28	35	75	398.76	286.48	51.72	46.67	41.37	420.15	291.17	56.39	52.79	47.56	399	286	51.7	46.7	41.4	643.54	411.64	76.84	71.12	66.30
17	0.5	70	28	35	65	656.45	412.92	77.54	73.85	68.67	619.4	400.83	75.90	72.02	67.17	656	413	77.5	73.9	68.7	572.68	346.90	66.05	62.82	57.70
18	0.75	65	23	51.89	70	556.34	366.45	69.6	65.73	61.32	553.18	366.19	69.89	65.54	60.56	556	366	69.6	65.7	56.1	572.68	346.90	66.05	62.82	57.70
19	0.75	65	23	40	70	482.67	325.67	72.74	68.63	63.65	483.67	324.5	72.28	67.22	62.85	484	325	72.5	67.5	63.1	643.54	411.64	76.84	71.12	66.30
20	0.75	65	34.89	40	70	536.32	367.34	69.43	65.91	61.27	559.71	360	66.64	63.10	58.07	536	367	69.4	65.9	61.3	631.10	393.11	74.63	68.02	63.66
21	1	60	18	45	75	404.45	278.31	56.71	51.76	46.64	447.98	309.3	62.20	58.14	53.02	404	278	56.7	51.8	46.6	643.54	411.64	76.84	71.12	66.30
22	1	60	28	45	65	395.67	264.37	53.56	47.62	42.27	403.45	277.84	54.12	49.77	44.72	396	264	53.6	47.7	42.3	572.68	346.90	66.05	62.82	57.70
23	0.75	65	23	40	70	481.36	326.54	74.75	70.74	64.18	483.67	324.5	72.28	67.22	62.85	484	325	72.5	67.5	63.1	631.10	393.11	74.63	68.02	63.66
24	0.5	70	28	45	65	654.34	432.64	78.56	74.74	70.46	626.1	412.88	72.99	69.27	64.96	654	433	78.6	74.7	70.5	643.54	411.64	76.84	71.12	66.30
25	1	70	28	35	65	422.53	293.08	58.93	53.41	48.45	419.15	276.39	57.47	52.18	47.54	423	293	58.9	53.4	48.4	417.93	301.03	61.55	57.98	53.38
26	0.75	65	23	40	81.892	602.37	399.45	73.72	69.86	65.78	573.3	379.18	69.72	65.10	60.9	602	399	73.7	69.9	65.8	572.68	346.91	66.05	62.82	57.70
27	0.75	65	11.11	40	70	574.32	348.52	68.56	65.82	60.43	544.66	349.29	68.60	64.88	60.04	574	349	68.6	65.8	60.4	631.10	393.11	74.63	68.02	63.66
28	0.75	65	23	40	70	484.65	324.07	71.85	64.74	59.78	483.67	324.5	72.28	67.22	62.85	484	325	72.5	67.5	63.1	631.10	393.11	74.63	68.02	63.66
29	0.5	70	18	35	75	562.12	371.74	73.54	67.84	63.28	575.57	376.85	70.90	66.44	61.67	562	372	73.5	67.8	63.3	631.10	393.11	74.63	68.02	63.66
30	0.75	65	23	40	70	485.34	325.94	69.93	65.34	62.54	483.67	324.5	72.28	67.22	62.85	484	325	72.5	67.5	63.1	572.68	346.90	66.05	62.82	57.70
31	0.5	60	28	35	75	660.12	418.6	79.45	74.93	70.52	624.86	406.96	76.26	71.99	67.21	660	419	79.4	74.9	70.5	631.10	393.11	74.63	68.02	63.66
32	0.5	70	18	45	65	641.32	401.83	75.34	73.32	68.56	627.62	405.62	76.03	72.93	68.37	641	402	75.3	73.3	68.6	643.54	411.64	76.84	71.12	66.30
33	0.5	70	28	35	75	633.35	401.162	72.23	70.89	66.28	616.9	406.52	77.16	74.04	69.19	633	401	72.2	70.9	66.3	631.10	393.11	74.63	68.02	63.66
34	1	70	28	45	65	421.65	274.92	55.63	51.62	47.31	437.91	299.48	60.36	55.51	51.13	422	275	55.6	51.6	47.3	631.10	393.11	74.63	68.02	63.66
35	0.5	60	18	35	65	636.45	392.43	74.98	67.56	63.45	660.13	403.42	78.5	72.92	67.96	636	392	75	67.6	63.5	643.54	411.64	76.84	71.12	66.30
36	0.75	53.11	23	40	70	586.05	348.65	64.76	61.43	56.34	540.82	345.43	62.22	57.88	52.87	586	349	64.8	61.4	56.3	572.68	346.90	66.05	62.82	57.70
37	1	70	18	35	65	418.9	268.06	53.78	48.04	44.36	427.11	291.69	58.33	53.76	50.01	419	268	53.8	48	44.4	643.54	411.64	76.84	71.12	66.30
38	0.75	65	23	40	70	485.76	323.54	75.89	70.05	66.88	483.67	324.5	72.28	67.22	62.85	484	325	72.5	67.5	63.1	643.54	411.64	76.84	71.12	66.30
39	0.5	70	28	45	75	645.34	404.72	77.84	69.42	65.24	666.48	431.18	80.95	76.42	72.43	645	405	77.8	69.4	65.2	631.10	393.11	74.63	68.02	63.66
40	1	70	28	35	75	423.76	278.43	55.58	51.56	46.65	449.81	301.07	57.05	53.74	48.43	424	278	55.6	51.6	46.7	417.93	301.03	61.55	57.98	53.38
41	0.75	65	23	40	70	483.72	324.65	72.86	66.83	62.41	483.67	324.5	72.28	67.22	62.85	484	325	72.5	67.5	63.1	417.93	301.03	61.55	57.98	53.38
42	0.5	60	18	45	75	638.34	401.61	76.45	69.49	65.56	630.09	391.51	75.03	68.95	64.16	638	402	76.4	69.5	65.6	572.68	346.90	66.05	62.82	57.70
43	1.35	65	23	40	70	360.76	243.17	50.51	45.76	41.07	286.35	221.78	46.18	41.73	36.48	361	243	50.5	45.8	41.1	643.54	411.64	76.84	71.12	66.30
44	0.75	65	23	40	58.11	534.63	345.92	66.76	62.56	58.26	557.44	359.62	68.01	63.56	59.54	535	346	66.8	62.6	58.3	643.54	411.64	76.84	71.12	66.30
45	0.75	65	23	40	70	485.65	325.73	71.03	68.45	64.07	483.67	324.5	72.28	67.22	62.85	484	325	72.5	67.5	63.1	572.68	346.90	66.05	62.83	57.70
46	0.75	65	23	40	70	484.38	324.23	70.98	65.43	61.68	483.67	324.5	72.28	67.22	62.85	484	325	72.5	67.5	63.1	417.93	301.02	61.55	57.98	53.38
47	1	60	28	35	65	396.06	256.34	54.76	50.56	45.56	522.2	334.46	67.52	62.47	57.34	396	256	54.8	50.6	45.6	631.10	393.11	74.63	68.02	63.66
48	1	60	18	35	75	380.76	268.04	52.56	47.58	43.34	497.88	332.57	65.7	60.41	55.77	381	268	52.6	47.6	43.3	417.93	301.02	61.55	57.98	53.38
49	0.5	60	28	45	75	642.82	402.63	73.67	69.67	65.34	650.8	415.85	74.23	69.04	64.97	643	403	73.7	69.7	65.3	643.54	411.64	76.84	71.12	66.30
50	0.5	60	28	35	65	636.42	393.95	74.76	68.72	63.34	646.2	405.29	75.24	70.51	65.12	636	394	74.8	68.7	63.3	631.10	393.11	74.63	68.02	63.66

*All the experiments repeated three times.

The above Eq. (5) could be converted, which is given below based on the value of five variables,

$${\text{Y}} = \alpha_{0} + \alpha_{{1}} {\text{X}}_{{1}} + \alpha_{{2}} {\text{X}}_{{2}} + \alpha_{{3}} {\text{X}}_{{3}} + \alpha_{{4}} {\text{X}}_{{4}} + \alpha_{{5}} {\text{X}}_{{{5} + }} \alpha_{{{12}}} {\text{X}}_{{1}} {\text{X}}_{{2}} + \alpha_{{{13}}} {\text{X}}_{{1}} {\text{X}}_{{3}} + \alpha_{{{14}}} {\text{X}}_{{1}} {\text{X}}_{{4}} + \alpha_{{{15}}} {\text{X}}_{{1}} {\text{X}}_{{5}} + \alpha_{{{23}}} {\text{X}}_{{2}} {\text{X}}_{{3}} + \alpha_{{{24}}} {\text{X}}_{{2}} {\text{X}}_{{4}} + \alpha_{{{25}}} {\text{X}}_{{2}} {\text{X}}_{{5}} + \alpha_{{{34}}} {\text{X}}_{{3}} {\text{X}}_{{4}} + \, \alpha_{{{35}}} {\text{X}}_{{3}} {\text{X}}_{{5}} + \alpha_{{{45}}} {\text{X}}_{{4}} {\text{X}}_{{5}} + \alpha_{{{11}}} {\text{X}}_{{1}}^{{2}} + \alpha_{{{22}}} {\text{X}}_{{2}}^{{2}} + \, \alpha_{{{33}}} {\text{X}}_{{3}}^{{2}} + \alpha_{{{44}}} {\text{X}}_{{4}}^{{2}} + \alpha_{55} X_{5}^{2} ,$$

where *Y* is the dependent response, α_0_ is the coefficients-constant of the intercept, αi is linear, αii is quadratic, and αij are interaction terms. *X*i and *X*j are coded values of independent variables of particle size (*X*_1_), methanol concentration (*X*_2_), ultrasound exposure time (*X*_3_), temperature (*X*_4_), and ultrasound intensity (*X*_5_), and ε is an error. Model significance (*p* value), coefficient of determination (R^2^), predicted coefficient of determination (R^2^ pred), adjusted coefficient of determination (R^2^ adj), and the adequacy of the models by the statistic lack-of-fit value were all determined by analysis of variance (ANOVA). Only significant coefficients (*p* < 0.05) or those necessary for the hierarchy were considered when creating the models. Further analysis was performed to determine the accuracy of the extraction parameters.

### Modeling an adaptive neuro-fuzzy inference system (ANFIS) for optimization

Adaptive neuro‑fuzzy inference system (ANFIS) has an advantage over artificial neural networks (ANN) because it combines the best features of neural networks and fuzzy logic to model complex systems more accurately and precisely^52^. They are inspired by the properties of biological neural networks that resemble the human brain; these networks learn from experience and are used in data processing for categorization and prediction^53^. It is also suitable for various applications, such as optimizing significant extraction variables, thanks to its ability to analyse both numerical and linguistic data^54^. Additionally, merging ANN with fuzzy-set theory helps address the benefits and limitations of both approaches^55^. Jang can develop the intelligent computer tool ANFIS, which can be used to solve complex and nonlinear issues^56^. Both linear and nonlinear relationships between input and output responses can be analysed with this method^57^. This method employs a rule-based fuzzy logic model, which is trained with the help of rules generated during the procedure^58^. Data is used to inform the training process^59^. Furthermore, training datasets are provided by least squares and backpropagation modeling in this system. Backpropagation of ANN is used as the first step in training data for the adaptive network-based fuzzy inference system (ANFIS)^60^. The output response of ANN will then be used to fuzzy logic membership functions as the input parameters of particle size (*X*_1_), methanol concentration (*X*_2_), ultrasound exposure time (*X*_3_), temperature (*X*_4_), and ultrasound intensity (*X*_5_). These variable optimizations are performed with greater precision thanks to the fuzzy inference system (FIS). The backpropagation algorithm is employed as the initial training strategy in the adaptive network-based fuzzy inference system (ANFIS) for data training purposes. The input parameters of particle size (*X*_1_), methanol concentration (*X*_2_), ultrasound exposure time (*X*_3_), temperature (*X*_4_), and ultrasound intensity (*X*_5_) will be utilized as input variables for the artificial neural network (ANN). The output response of the ANN will subsequently be employed in the application of fuzzy logic membership functions. Utilizing the fuzzy inference system (FIS) enhances the accuracy of the optimizations pertaining to these variables. The analysis of each predicted output responses of yield of TPC, TFC and percentage antioxidant scavenging potential (DPPH*sc, ABTS*sc, and FRAP) was done using the optimization of ANFIS modeling and data from similar CCD of RSM experiments.

**Figure 1:**
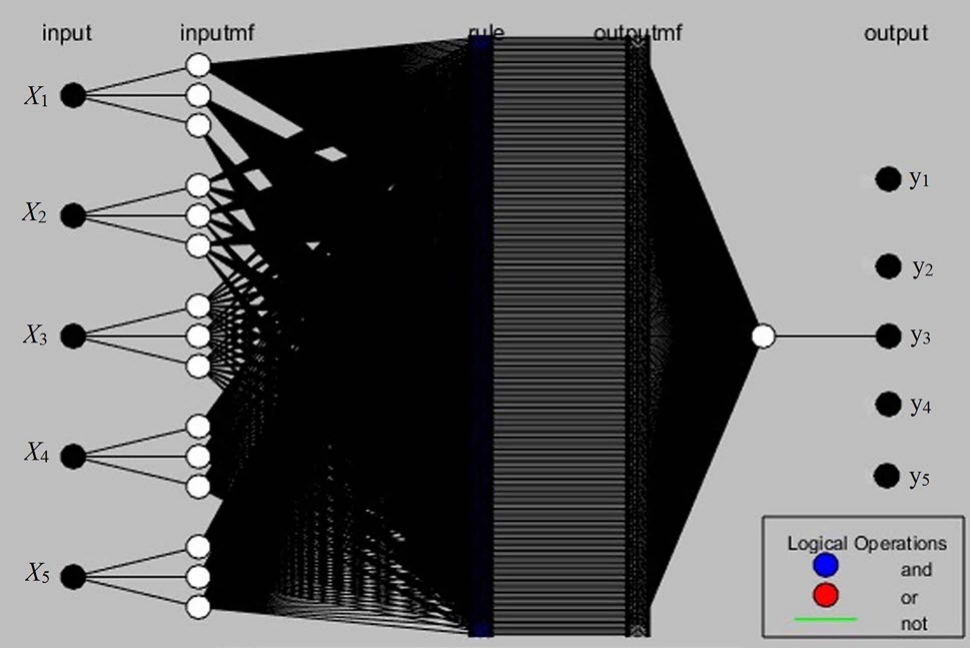
The architecture of the ANFIS input and output response model.

In this study, the Sugeno-type fuzzy inference model was employed for the ANFIS modeling to get multiple inputs (*X*_1_, *X*_2_, *X*_3_, *X*_4_, and *X*_5_) and a single output response (y1/y2/y3/y4/y5). Because the Sugeno-type fuzzy inference system is more computationally efficient than the Mamdani type. The Mamdani type depends more on specialized knowledge. Nonetheless, actual data is used to train the Sugeno type. The ANFIS architecture (Fig. 1) shows the design displayed multiple inputs and a single output response at a time. A Sugeno-Fuzzy Inference System (FIS) has one output response, “z,” and two inputs, “x” and “y”. Two fuzzy if–then rules for a first-order Sugeno fuzzy model can be expressed as follows:

- Rule 1: If x is A_1_ and y is B_1_, then f_1_ = p_1_x + q_1_y + r_1_,
- Rule 2: If x is A_2_ and y is B_2_, then f_2_ = p_2_x + q_2_y + r_2_,

where A_1_ and B_1_ are the fuzzy sets, f_1_ is the output response, and p_1_, q_1_, and r_1_ are the design parameters determined during the training process. The number of membership functions for each given input variable was determined by a procedure of trial and error. To predict the outcome of the extraction of the majority of bioactive components from grape seeds extract, the experimental data was divided into training, testing, and validation of the network model using MATLAB v. R2013a Fuzzy logic toolbox.

### Optimization using machine learning algorithm

The technique of continuously increasing a machine learning model’s accuracy and decreasing its error rate is known as machine learning optimization^61^. Most machine learning models use training data to understand the link between input and output responses. After this, the models can be applied to categorize fresh incoming data or predict trends. Since the target values of the experiment are continuous, a Random Forest Regressor is employed^62^. This ensemble learning approach combines different decision trees to create a more accurate model^63^. A subset of the training data and a randomly selected subset of the characteristics are used to train each decision tree. This randomization helps to improve the model’s prediction accuracy and reduce overfitting. The Random Forest algorithm generates many decision trees and then aggregates their forecasts to obtain a more accurate and reliable prediction^64^. The primary benefit of the random forest regressor is its ability to handle high-dimensional data with a nonlinear relationship between the input and target values. The data set for this investigation has experimental values* X*_1_, *X*_2_, *X*_3_, *X*_4_, and *X*_5_ as inputs and y_1_, y_2_, y_3_, y_4_, and y_5_ as output responses. In order to generate the predictions, the experimental values are the dataset that is initially imported. It is then preprocessed to see if there are any missing or noisy data in the dataset.

### Verification optimized condition

By evaluating the dependability of the optimization findings, the applicability of the experiment was confirmed. Under optimal conditions, a triplicate experiment was carried out based on the combination of response and minor deviation. The mean experimental results were compared to assess the model’s reasonableness about the predicted values.

### Utilizing GC–MS for volatile bioactive compounds identification

Gas chromatography and mass spectrometry (GC–MS) was used to determine the volatile nature of the bioactive components in the optimized grape seed extract^7^. The GC–MS analysis was performed in gas chromatography that also served as a mass spectrophotometer (Shimadzu Make QP-2010 GC–MS system), equipped with a non-polar 60 M RTX 5MS column and helium gas as the carrier gas, with a constant pressure of 15 psi and an adjusted column flow velocity of 1.00 mL × min^−1^ with initial oven temperature at 40 °C held for 3 min and the final temperature of the oven was 480 °C. with the rate at 10 °C [min × sup^−1^]. A 2 μL sample was injected with split-less mode. Mass spectra was recorded over 35–650 amu range with electron impact ionization energy 70 eV. The total running time for a sample is 45 min. Identification of bioactive ingredients was achieved based on their retention time of chromatographic peaks utilizing a Quadrapole detector and the NIST 2014 (National Institute of Standards and Technology, 2014) library to relative retention indices. NIST library database contains more than 62,000 patterns of well-known compounds. The spectra of the unknown bioactive ingredients of grape seeds extract fraction obtained were compared to the reference mass spectra of recognised components deposited in the NIST library collection (NIST).

### Utilizing LC–MS for non-volatile bioactive compounds identification

Liquid chromatography and mass spectrometry (LC–MS) was used to determine the non-volatile nature of the bioactive components in the optimized grape seed extract. The LC–MS analysis was performed using the 1290 Infinity UHPLC System, and 6550 iFunnel Q-TOFs (Agilent Technologies, USA). For separations Zorbax-SB-C-18 column (2.1 × 50 mm, 1.8 µM particle size, Agilent Technologies, USA). Two mobile phases were used: A-0.1% formic acid in water and B-90% of acetonitrile in water, at a flow rate of 500 µL min^−1^. The LC conditions were maintained at 5% at 0–3 min in B, a linear increase from 5 to 20% between 3 and 25 min, 20 to 40% during 25–40 min, and from 40 to 50% between 40 and 55 min, finally, it reached 50 to 95% at 55–63 min. The peak detection was performed through direct injection mode with an Electron Spray Ionization (ESI) probe, both positive and negative modes. The non-volatile nature of bioactive compounds was identified by obtaining the molecular mass and structural formula of compounds with the help of online libraries.

### Statistical analysis

All the experiments were performed based on RSM’s CCD and repeated three times. Design expert software (trial version 8.0.7.1, Stat-Ease, Inc., 2021 East Hennepin Ave, Suite 480, Minneapolis, MN 55413, USA) used for the experimental design, optimization, data analysis, prediction, and quadratic model building. In regression model, the goodness of fit was evaluated based on the R^2^ (coefficients of determination). Further, the statistical analysis was assessed by one-way analysis of variance (ANOVA), with *p*-values less than 0.05 considered significant. The optimal extraction conditions were analysed by contour plots and three-dimensional (3D) response surface plots. Microsoft Excel (Microsoft Office Professional Plus 2021) was used for statistical analysis and the Adaptive neuro-fuzzy logic toolbox in the MATLAB (Mathematical Laboratory) v R2013b software.

## Results and discussion

### Adequacy of the models

Ultrasound-aided extraction is one of the best techniques for extracting thermosensitive and minute bioactive ingredients from natural sources. This ultrasound-aided extraction delivers numerous advantages, such as consuming less solvent as well as energy for extraction, heat not generated during ultrasonic waves rupturing the cell wall, and ultrasonic waves very quickly breaking the cell wall and solubilizing the internal active ingredients by the solvent^65^. This study successfully optimized independent extraction variables through the CCD of RSM. This quadratic model was employed by combining ultrasound-aided extraction parameters of linear, interactions, and quadratic impacts on grape seed extract’s maximal extraction yield of bioactive compounds^66^. CCD was flexible and effective, and could provide much information about experimental variables and errors with the least experimental cycle^67^. Therefore, several experiments were carried out according to the central experiment design (CCD). Table 2 presented the experimental values and their predicted TPC, TFC, and antioxidant scavenging potentials (%DPPH*sc, %ABTS*sc, and FRAP) values of grapes seed extract under combination extraction parameters. Based on the experimental results, the optimized condition was observed at 0.155 mm particle size (*X*_1_), 65% methanol concentration (*X*_2_), 23 min ultrasound exposure time (*X*_3_), temperature (*X*_4_) at 40 °C, and ultrasound intensity (*X*_5_) was 70 W cm^−2^. This situation, the optimal yields of TPC, TFC, and antioxidant scavenging potential were achieved to be 670.32 mg GAE/g, 451.45 mg RE/g, 81.23% DPPH*sc, 77.39% ABTS*sc and 71.55 μg mol (Fe(II))/g FRAP value (Raw data was presented in Related file). The interaction of extraction parameters may be the primary factor causing the maximum production of bioactive components from grape seeds. Similar results were obtained in our previous optimization experiments, such as bioactive compounds from *Garcinia indica,* and *Hemidesmus indicus* Linn. of ultrasound-assisted extraction^7,14^. The experimental results fitted to the model of a second-order polynomial equation. ANOVA was used to analyse the regression equation that was obtained.

The significance of the coefficients was ascertained at a 95% confidence level using the F and *p* tests. The associated variables would become highly significant if the *p*-value decreases and the F-value increases^68^. The* p*-values were used as an essential tool to check the significance of the interactions of the variables^69^. Importantly, when the* p*-value was < 0.05, then the model terms are assigned as statistically significant. While, the *p*-value was greater than 0.05 the model terms are called non-significant. The obtained F value for the lack of fit in this investigation was 18.49, and the present model was therefore highly significant (*p*-value 0.0001 and F value 18.49). The multiple regression coefficient of determination (R^2^) determines the model’s output response and the importance of lack-of-fit. The adequacy-output response model revealed that the quadratic model multiple regression coefficient of determination (R^2^) of TPC, TFC, and antioxidant scavenging potentials (DPPH*sc, ABTS*sc, and FRAP) were 0.9273, 0.9323, 0.9045, 0.8730, and 0.8800, respectively, which demonstrated good depiction of the variables by the model and satisfied Le Man et al. For this reason, a model is considered acceptable when R^2^ > 0.87. The predicted R^2^ value (0.6930) is close to the adjusted R^2^ value (0.8772), and the 95% confidence level shows that the quadratic models fit the experimental data well. This implied that 95% of the experimental values agree with the model’s predictions.

### Investigation of the response surfaces

#### Total phenolic content (TPC)

The experimental results and their predicted values of TPC using various combinations of extraction parameters in the ultrasound-aided solvent extraction method are presented in Table 3. Using the obtained experimental data, an analysis of variance (ANOVA) was performed to determine the coefficient of determination (R^2^) of the model’s significance. The statistical significance of the model equation was evaluated using the lack-of-fit test, coefficient of determination (R^2^), and *p*-values^70,71^. From the analyzed data in Table 3 and polynomial Eq. (7), it was determined that the linear term of particle size has a substantial (*p* 0.05) contribution to the most significant yield of total phenolic content (*X*_1_), temperature (*X*_4_), and quadratic term *X*_2_^2^, *X*_3_^2^, *X*_5_^2^. As the ANOVA result in this model illustrates in Table 3, the model could reflect the relationship between the experimental values and their predicted responses with a higher F-value (18.49), and a very low probability value (*p* < 0.0001). Additionally, sufficient precision and the coefficient of determination (R^2^) were significant markers of the model fitting. An R^2^ value near one indicates that the suggested model provides a better explanation for the variability of the experimental data; in other words, there is a stronger correlation between the observed and predicted values^72^. The coefficient of determination of the ultrasound-aided solvent extraction of total polyphenolics was found to be R^2^ = 0.9273, R^2^ predicted = 0.6930, and R^2^ adjusted = 0.8772, which indicated that this model has good reliability and fitting. The second-order polynomial equation for the fitted quadratic model for TPC in coded variables is given below Eq. (6)

6 $$\begin{aligned} {\text{TPC }}\left( {{\text{y}}_{{1}} } \right) \, & = { 483}.{67} - {95}.{59}X_{{1}} + {2}.{5}X_{{2}} + {3}.{19}X_{{3}} + {12}.{2}X_{{4}} + {3}.{36}X_{{5}} + {9}.{4}X_{{1}} X_{{2}} - {2}.{64}X_{{1}} X3 + {3}.0{1}X_{{1}} X_{{4}} \\ &\quad + { 8}.{29}X_{{1}} X_{{5}} + {4}.{13}X_{{2}} X_{{3}} + {5}.{91}X_{{2}} X_{{4}} + {4}.{71}X_{{2}} X_{{5}} - {1}.0{3}X_{{3}} X_{{4}} + {9}.{69}X_{{3}} X_{{5}} + {1}0.{72}X_{{4}} X_{{5}} \\ &\quad + {5}.0{7}X_{{1}}^{{2}} + {11}.{14}X_{{2}}^{{2}} + {12}.{22}X_{{3}}^{{2}} + {7}.{16}X_{{4}}^{{2}} + {14}.{44}X_{{5}}^{{2}} . \end{aligned}$$

**Table 3:** Analysis of variance (ANOVA) for the quadratic polynomial mode.

Source	Sum of squares	df^a^	Mean square	F-value^b^	p-value^c^
TPC (y_1_)^d^					
Model	439,900	20	21,993.66	18.49	< 0.0001
*X*_1_	395,800	1	3.96E+05	332.8	< 0.0001
*X*_2_	270.07	1	270.07	0.2271	0.6373
*X*_3_	440.06	1	440.06	0.37	0.5477
*X*_4_	6447.81	1	6447.81	5.42	0.0271
*X*_*5*_	489.66	1	489.66	0.4118	0.5261
*X*_1_*X*_2_	2830.15	1	2830.15	2.38	0.1337
*X*_1_*X*_3_	222.29	1	222.29	0.1869	0.6687
*X*_1_*X*_4_	290.77	1	290.77	0.2445	0.6247
*X*_1_*X5*	2198.84	1	2198.84	1.85	0.1844
*X*_2_*X*_3_	545	1	545	0.4583	0.5038
*X*_2_*X*_4_	1117.46	1	1117.46	0.9397	0.3404
*X*_3_*X*_4_	710.46	1	710.46	0.5974	0.4458
*X*_3_*X*_5_	33.74	1	33.74	0.0284	0.8674
*X*_4_*X*_5_	3002.74	1	3002.74	2.53	0.1229
*X*_1_^2^	3677.82	1	3677.82	3.09	0.0892
*X*_*2*_^2^	1430.88	1	1430.88	1.2	0.2817
*X*_3_^2^	6893.43	1	6893.43	5.8	0.0226
*X*_4_^*2*^	8144.72	1	8144.72	6.85	0.0139
*X*_5_^*2*^	2849.08	1	2849.08	2.4	0.1325
Residual	11,581.3	1	11,581.28	9.74	0.0041
Lack of fit	34,486.8	29	1189.2		
Pure error	34,470.1	22	1566.82	656.48	< 0.0001
Cor total	16.71	7	2.39		
TFC (y_2_)^e^					
Model	129,800	20	6491.91	19.96	< 0.0001
*X*_1_	115,600	1	1.16E+05	355.45	< 0.0001
*X*_2_	679.09	1	679.09	2.09	0.1592
*X*_3_	221.21	1	221.21	0.6801	0.4163
*X*_4_	2763.7	1	2763.7	8.5	0.0068
*X*_*5*_	737.91	1	737.91	2.27	0.1428
*X*_1_*X*_2_	213.72	1	213.72	0.657	0.4242
*X*_1_*X*_3_	1015	1	1015	3.12	0.0878
*X*_1_*X*_4_	243.55	1	243.55	0.7488	0.394
*X*_1_*X5*	720.84	1	720.84	2.22	0.1474
*X*_2_*X*_3_	57.46	1	57.46	0.1767	0.6774
*X*_2_*X*_4_	496.9	1	496.9	1.53	0.2264
*X*_3_*X*_4_	32.37	1	32.37	0.0995	0.7547
*X*_3_*X*_5_	0.0021	1	0.0021	6.40E-06	0.998
*X*_4_*X*_5_	1006.91	1	1006.91	3.1	0.0891
*X*_1_^2^	317.76	1	317.76	0.9769	0.3311
*X*_*2*_^2^	660.42	1	660.42	2.03	0.1649
*X*_3_^2^	1594.97	1	1594.97	4.9	0.0348
*X*_4_^*2*^	1575.56	1	1575.56	4.84	0.0359
*X*_5_^*2*^	894.54	1	894.54	2.75	0.108
Residual	3497.2	1	3497.2	10.75	0.0027
Lack of fit	9432.79	29	325.27		
Pure error	9424.85	22	428.4	378.08	< 0.0001
Cor total	7.93	7	1.13		
%DPPH*sc (y_3_)^f^					
Model	3314.9	20	165.74	13.73	< 0.0001
*X*_1_	2645.01	1	2645.01	219.12	< 0.0001
*X*_2_	33.29	1	33.29	2.76	0.1076
*X*_3_	7.45	1	7.45	0.617	0.4385
*X*_4_	49.66	1	49.66	4.11	0.0518
*X*_*5*_	5.49	1	5.49	0.455	0.5053
*X*_1_*X*_2_	0.1164	1	0.1164	0.0096	0.9224
*X*_1_*X*_3_	3.06	1	3.06	0.2532	0.6186
*X*_1_*X*_4_	67.48	1	67.48	5.59	0.025
*X*_1_*X5*	5.64	1	5.64	0.4669	0.4998
*X*_2_*X*_3_	26.44	1	26.44	2.19	0.1496
*X*_2_*X*_4_	67.77	1	67.77	5.61	0.0247
*X*_3_*X*_4_	0.1093	1	0.1093	0.0091	0.9249
*X*_3_*X*_5_	23.38	1	23.38	1.94	0.1746
*X*_4_*X*_5_	69.24	1	69.24	5.74	0.0233
*X*_1_^2^	90.01	1	90.01	7.46	0.0106
*X*_*2*_^2^	105.14	1	105.14	8.71	0.0062
*X*_3_^2^	110.2	1	110.2	9.13	0.0052
*X*_4_^*2*^	37.65	1	37.65	3.12	0.0879
*X*_5_^*2*^	42.31	1	42.31	3.51	0.0713
Residual	20.21	1	20.21	1.67	0.2059
Lack of fit	350.06	29	12.07		
Pure error	321.82	22	14.63	3.63	0.0431
Cor total	28.24	7	4.03		
%ABTS*sc (y_4_)^g^					
Model	3196.49	20	159.82	9.97	< 0.0001
*X*_1_	2543.85	1	2543.85	158.7	< 0.0001
*X*_2_	58.62	1	58.62	3.66	0.0658
*X*_3_	6.13	1	6.13	0.3825	0.5411
*X*_4_	61.36	1	61.36	3.83	0.0601
*X*_*5*_	4.51	1	4.51	0.2812	0.6
*X*_1_*X*_2_	2.43	1	2.43	0.1513	0.7001
*X*_1_*X*_3_	10.66	1	10.66	0.6651	0.4214
*X*_1_*X*_4_	73.84	1	73.84	4.61	0.0403
*X*_1_*X5*	0.4209	1	0.4209	0.0263	0.8724
*X*_2_*X*_3_	19.58	1	19.58	1.22	0.2782
*X*_2_*X*_4_	56.9	1	56.9	3.55	0.0696
*X*_3_*X*_4_	0.5913	1	0.5913	0.0369	0.849
*X*_3_*X*_5_	38.43	1	38.43	2.4	0.1324
*X*_4_*X*_5_	94.57	1	94.57	5.9	0.0216
*X*_1_^2^	52.56	1	52.56	3.28	0.0806
*X*_*2*_^2^	98.26	1	98.26	6.13	0.0194
*X*_3_^2^	74.67	1	74.67	4.66	0.0393
*X*_4_^*2*^	18.14	1	18.14	1.13	0.2961
*X*_5_^*2*^	35.28	1	35.28	2.2	0.1487
Residual	14.48	1	14.48	0.9032	0.3498
Lack of fit	464.86	29	16.03		
Pure error	428.67	22	19.48	3.77	0.039
Cor total	36.19	7	5.17		
FRAP (y_5_)^h^					
Model	3282.89	20	164.14	10.64	< 0.0001
*X*_1_	2564.2	1	2564.2	166.17	< 0.0001
*X*_2_	76.61	1	76.61	4.96	0.0338
*X*_3_	7.49	1	7.49	0.4854	0.4915
*X*_4_	67.35	1	67.35	4.36	0.0456
*X*_*5*_	3.5	1	3.5	0.2269	0.6374
*X*_1_*X*_2_	2.48	1	2.48	0.1608	0.6914
*X*_1_*X*_3_	14.62	1	14.62	0.9475	0.3384
*X*_1_*X*_4_	67.31	1	67.31	4.36	0.0456
*X*_1_*X5*	2.57	1	2.57	0.1666	0.6862
*X*_2_*X*_3_	18.93	1	18.93	1.23	0.2772
*X*_2_*X*_4_	59.98	1	59.98	3.89	0.0583
*X*_3_*X*_4_	0.0109	1	0.0109	0.0007	0.979
*X*_3_*X*_5_	26.48	1	26.48	1.72	0.2005
*X*_4_*X*_5_	106.32	1	106.32	6.89	0.0137
*X*_1_^2^	59.32	1	59.32	3.84	0.0596
*X*_*2*_^2^	120.83	1	120.83	7.83	0.009
*X*_3_^2^	80.57	1	80.57	5.22	0.0298
*X*_4_^2^	25.1	1	25.1	1.63	0.2123
*X*_5_^2^	48.08	1	48.08	3.12	0.0881
Residual	12.03	1	12.03	0.7794	0.3846
Lack of fit	447.5	29	15.43		
Pure error	416.99	22	18.95	4.35	0.0264
Cor total	30.51	7	4.36		

^a^Degrees of freedom.

^b^Test for comparing model variance with residual (error) variance.

^c^Probability of seeing the observed F value if the null hypothesis is true.

^d^Std Dev: 34.48; Mean: 526.91; R^2^ = 0.9273; R^2^ predicted = 0.6930; R^2^ adjusted = 0.8772.

^e^Std Dev: 18.04; Mean: 347.09; R^2^ = 0.9323; adjusted R^2^ = 0.8856; predicted R^2^ = 0.7567.

^f^Std Dev: 3.47; Mean: 67.88; R^2^ = 0.9045; adjusted R^2^ = 0.8386; predicted R^2^ = 0.6661.

^g^Std Dev: 4.0; Mean: 63.43; R^2^ = 0.8730; adjusted R^2^ = 0.7855; predicted R^2^ = 0.5611.

^h^Std Dev: 3.93; Mean: 58.74; R^2^ = 0.8800; adjusted R^2^ = 0.7973; predicted R^2^ = 0.5789.

**Figure 2:**
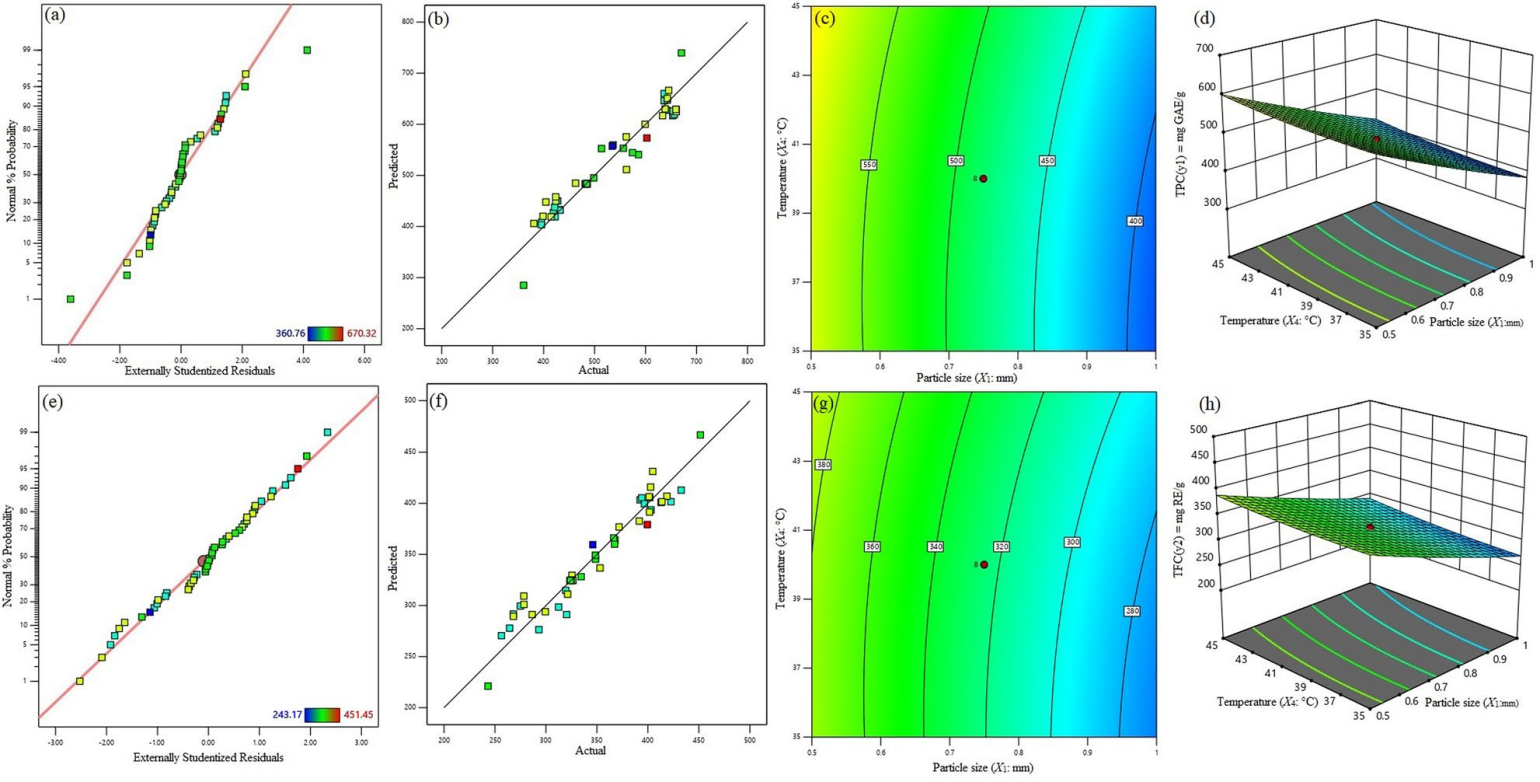
Normal percentage probability plot for the studentized residuals for highest yield of TPC (**a**), and TFC (**e**). Relationship between experimental and predicted value for highest yield of TPC (**b**), and TFC (**f**), Response surface and contour plot showing the combined effects of methanol concentration (*X*_1_) and temperature (*X*_2_) for highest yield of TPC, and TFC, when time and particle size were held at fixed level (**c,g,d,h**), respectively.

The residuals were subsequently examined using the model data. Using residuals, it was possible to determine the difference between an experimental value from a response surface measurement and the value that the model anticipated. Figure 2a displays the studentized residuals of *X*_1_, *X*_2_, *X*_3_, *X*_4_, and *X*_5_ as a normal percent probability plot. These discovered variations do not deviate from the usual distribution. A model that fits the data well is indicated by a high coefficient of determination (R^2^ >> 0.9), as seen in Fig. 2b. Figure 2c,d show 3D response surface and 2D contour plot reveal the significant effect of particle size (*X*_1_) and temperature (*X*_4_) in maximizing yield of TPC with methanol concentration, ultrasonic exposure time and ultrasonic intensity held at a fixed level (zero level) = 65%, 23 min, 70 W cm^−2^, respectively).The effects of particle size (*X*_1_), temperature (*X*_4_), and the extract’s highest content of total polyphenolic content was further illustrated by the 3D response surface and contour plot in Fig. 2c,d. The total polyphenolic concentrations ranged from 360.76 to 670.32 mg GAE/g, as Table 2 demonstrates. The maximum yield of total polyphenolic content was achieved at 0.155 mm particle size, 65% methanol concentration, 23 min ultrasonic exposure time, temperature at 40 °C, and 60 W cm^−2^ ultrasonic intensity.

#### Total flavonoid content (TFC)

From the ANOVA Table 3 and obtained second-order polynomial Eq. (8) illustrated that the linear term particle size (*X*_1_) and temperature (*X*_4_) and quadratic term *X*_2_^2^, *X*_3_^2^, and *X*_5_^2^ are influencing significant (*p* << 0.05) effects for the maximum extraction yield of total flavonoid content from grape seeds extract. The effect of other terms was found to be non-significant because *p* value was greater than 0.05. The experimental model successfully fits the data, as evidenced by the response surface analysis of the total flavonoid content of the extract, which revealed a high coefficient of determination value of R^2^ = 0.9323, adjusted R^2^ = 0.8856, predicted R^2^ = 0.7567 and a least value for lack of fit *p* value < 0.0001. Further, the adjusted R^2^ value close to predicted R^2^ value showed this model fitting one.

7 $$\begin{aligned} {\text{TFC}}\left( {{\text{y}}_{{2}} } \right) \, & = { 324}.{5} - {51}.{6659}X_{{1}} + {3}.{96}X_{{2}} + {2}.{26}X_{{3}} + {7}.{99}X_{{4}} + {4}.{13}X_{{5}} + {2}.{58}X_{{1}} X_{{2}} - {5}.{63}X_{{1}} X3\\ &\quad + {2}.{76}X_{{1}} X_{{4}} + { 4}.{75}X_{{1}} X_{{5}} + {1}.{34}X_{{2}} X_{{3}} + {3}.{94}X_{{2}} X_{{4}} + {1}.0{1}X_{{2}} X_{{5}} + 0.00{81}X_{{3}} X_{{4}} + {5}.{61}X_{{3}} X_{{5}} \\ &\quad + {3}.{15}X_{{4}} X_{{5}} + {3}.{45}X_{{1}}^{{2}} + {5}.{36}X_{{2}}^{{2}} + {5}.{32}X_{{3}}^{{2}} + {4}.0{1}X_{{4}}^{{2}} + {7}.{93}X_{{5}}^{{2}} . \end{aligned}$$

Figure 2e illustrates normal % probability plot of studentized residuals of *X*_1_, *X*_2_, *X*_3_, *X*_4_ and *X*_5_. These variants are normally distributed without any deviations. Coefficient of determination (R^2^) value should be close 0.9 to have a good fit of the model. The closer the goodness of fit to 1, the better the empirical model fits the actual data^73^. Figure 2f displayed the high coefficient of determination values (R^2^ >> 0.9), which are indicative of a strong fit. Furthermore, the maximum content of total flavonoids in the grapes seeds extract is influenced by particle size (*X*_1_) and temperature (*X*_4_). In contrast, other three variables, such as methanol concentration (*X*_2_), ultrasonic time (*X*_3_), and ultrasonic intensity (*X*_5_) were kept constant (zero level) = 65%, 23 min, 70 W cm^−2^, respectively, as shown by the 3D response surface and contour plot in Fig. 2g,h. Table 2 showed a range of 243.17 to 451.45 mg (RE)/g for total flavonoids. The highest yield of flavonoids was produced with 0.155 mm particle size, 65% methanol concentration, 23 min ultrasonic exposure time, temperature at 40 °C and 60 W cm^−2^ ultrasonic intensity; the lowest content was produced with 1.35 mm particle size, 65% methanol concentration, 23 min ultrasonic exposure time, temperature at 40 °C and 70 W cm^−2^ ultrasonic intensity.

### Antioxidant scavenging potentials (%DPPH*sc, %ABTS*sc and FRAP)

Based on the statistical analysis of experimental data in Table 3 and second-order polynomial Eqs. (8)–(10), the linear term *X*_1_ interaction terms *X*_1_*X*_4_, *X*_3_*X*_5_, and quadratic terms *X*_1_^2^, X_2_^2^are significantly (*p* < 0.05) contributing to the effects for the maximum yield of all three antioxidants (%DPPH*sc, %ABTS*sc, FRAP) scavenging potential from grape seeds extract. In addition, Table 3 shows the interaction terms *X*_2_*X*_4_, *X*_4_*X*_5_ contributing the highest DPPH* scavenging activity of grapes seeds extract. Similarly, linear term *X*_4_, and interaction term *X*_3_*X*_5_ has a significant effect on ABTS*sc and FRAP. The coefficient of determination (R^2^) value of %DPPH*sc, %ABTS*sc, and FRAP are 0.9045, 0.8730, 0.8800 respectively, adjusted R^2^ value of %DPPH*sc, %ABTS*sc, and FRAP are 0.8386, 0.7855, 0.7973 respectively, the predicted R^2^ value of %DPPH*sc, %ABTS*sc, and FRAP are 0.6661, 0.5611, 0.5789, respectively. All three antioxidant potentials adjusted R^2^ values very close to predicted R^2^, with the least lack of fit *p* value of %DPPH*sc, %ABTS*sc, and FRAP < 0.0001, < 0.0001, and < 0.0001, respectively. These observed data suggested that the model is significantly accurate. The second-order polynomial equation for the fitted quadratic models for %DPPH*sc, %ABTS*sc, and FRAP in coded variables is given in Eqs. (8)–(10).

8 $$\begin{aligned} \% {\text{DPPH}}*{\text{sc}}\left( {{\text{y}}_{{3}} } \right) \, & = { 72}.{28} - {7}.{81}X_{{1}} + 0.{8766}X_{{2}} - 0.{4147}X_{{3}} + {1}.0{7}X_{{4}} + 0.{3561}X_{{5}} - 0.0{6}0{3}X_{{1}} X_{{2}} \\ &\quad- 0.{3}0{91}X_{{1}} X3 + {1}.{45}X_{{1}} X_{{4}} - 0.{4197}X_{{1}} X_{{5}} + 0.{9}0{91}X_{{2}} X_{{3}} + {1}.{46}X_{{2}} X_{{4}} \\ &\quad+ 0.0{584}X_{{2}} X_{{5}} - 0.{8547}X_{{3}} X_{{4}} + {1}.{47}X_{{3}} X_{{5}} + { 1}.{68}X_{{4}} X_{{5}} - {1}.{38}X_{{1}}^{{2}} - {1}.{41}X_{{2}}^{{2}} \\ &\quad- 0.{8231}X_{{3}}^{{2}} - 0.{8726}X_{{4}}^{{2}} - 0.{6}0{3}0X_{{5}}^{{2}} , \end{aligned}$$

9 $$\begin{aligned} \% {\text{ABTS}}*{\text{sc}}\left( {{\text{y}}_{{4}} } \right) \, & = {67}.{22} - {7}.{66}X_{{1}} + {1}.{16}X_{{2}} - 0.{3762}X_{{3}} + {1}.{19}X_{{4}} + 0.{3226}X_{{5}} - 0.{2753}X_{{1}} X_{{2}} \\ &\quad- 0.{5772}X_{{1}} X3 + {1}.{52}X_{{1}} X_{{4}} - 0.{1147}X_{{1}} X_{{5}} + 0.{7822}X_{{2}} X_{{3}} + {1}.{33}X_{{2}} X_{{4}} \\ &\quad+ 0.{1359}X_{{2}} X_{{5}} - {1}.{1}0X_{{3}} X_{{4}} + {1}.{72}X_{{3}} X_{{5}} + {1}.{28}X_{{4}} X_{{5}} - { 1}.{33}X_{{1}}^{{2}} - { 1}.{16}X_{{2}}^{{2}} \\ &\quad- 0.{5714}X_{{3}}^{{2}} - 0.{7968}X_{{4}}^{{2}} - 0.{51}0{4}X_{{5}}^{{2}} , \end{aligned}$$

10 $$\begin{aligned} {\text{FRAP}}\left( {{\text{y}}_{{5}} } \right) \, & = { 62}.{85} - {7}.{69}X_{{1}} + {1}.{33}X_{{2}} - 0.{4159}X_{{3}} + {1}.{25}X_{{4}} + 0.{2843}X_{{5}} - 0.{2784}X_{{1}} X_{{2}} \\ &\quad- 0.{6759}X_{{1}} X_{{3}} + {1}.{45}X_{{1}} X_{{4}} - 0.{2834}X_{{1}} X_{{5}} + 0.{7691}X_{{2}} X_{{3}} + {1}.{37}X_{{2}} X_{{4}} \\ &\quad- 0.0{184}X_{{2}} X_{{5}} - 0.{9}0{97}X_{{3}} X_{{4}} + {1}.{82}X_{{3}} X_{{5}} + {1}.{36}X_{{4}} X_{{5}} - { 1}.{47}X_{{1}}^{{2}} - {1}.{2}0X_{{2}}^{{2}}\\ &\quad - 0.{672}0X_{{3}}^{{2}} - 0.{93}0{1}X_{{4}}^{{2}} - 0.{4652}X_{{5}}^{{2}} . \end{aligned}$$

**Figure 3:**
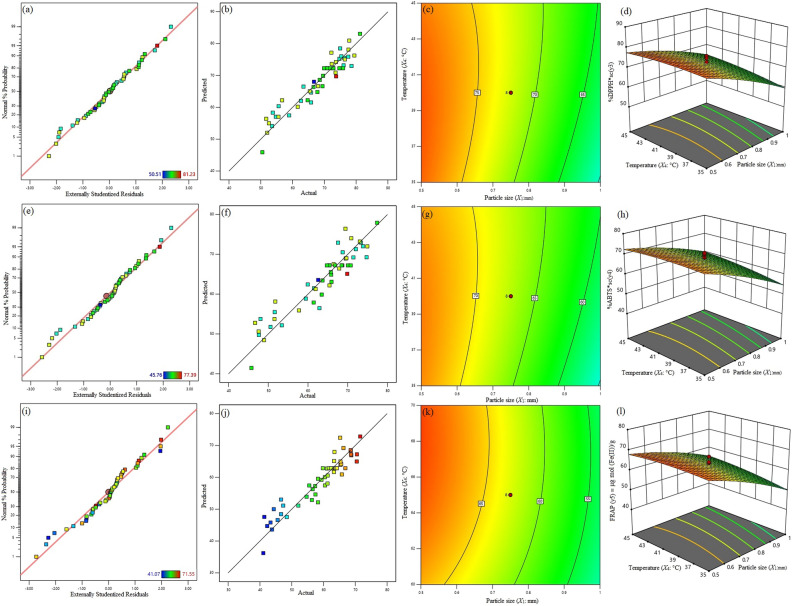
Normal percentage probability plot for the studentized residuals for highest yield of %DPPHsc (**a**), %ABTSsc (**e**) and FRAP (**i**). Relationship between experimental and predicted value for highest yield of %DPPHsc (**b**), %ABTSsc (**f**) and FRAP (**j**). Response surface and contour plot showing the combined effects of methanol concentration (*X*_1_) and temperature (*X*_2_) for highest yield of %DPPHsc, %ABTSsc and FRAP when time and particle size were held at fixed level (**c,g,k,d,h,l**), respectively.

Figure 3a,e,i shows that the normal percentage probability plot of studentized residuals of *X*_1_, *X*_2_, *X*_3_ and *X*_4_ and these variants are normally distributed and have no deviation for all three antioxidant scavenging experiments. Figure 3b,f,j displayed the high coefficient of determination (R^2^ >> 0.87), which are indicative of a strong fit. The 3D response surfaces and 2D contour plots for antioxidant scavenging potentials (%DPPH*sc, %ABTS*sc and FRAP) as responsible functional variables of particle size (*X*_1_) and temperature (*X*_4_) are shown in Fig. 3c,d,g,h,k,l. The figures show that 0.155 mm particle size, 65% methanol concentration, 23 min ultrasonic exposure time, temperature at 40 °C and 60 W cm^−2^ ultrasonic intensity correspond to the highest antioxidant (%DPPH*sc, %ABTS*sc, and FRAP) potential. The highest yields of antioxidant scavenging potentials are %DPPH 81.23%, %ABTS 77.39%, and FRAP 71.55 μg mol Fe (II)/g.

### ANFIS modelling

ANFIS modelling was used to investigate further verify experimental data and predict the extraction variables of bioactive ingredients in the grape seeds extract. The same 50 experimental data sets shown in Table 2 were divided into three sets to develop the ANFIS model prediction: 65% for the training data sets, 30% for the testing data sets, and 5% for validating the models. These sets were then used to construct a fuzzy inference system, the parameters of which were adjusted for the membership function using the least-squares method in conjunction with the back-propagation algorithm. The fuzzy logic toolbox in MATLAB v. R2013a was used to train ANFIS to obtain the results. To ensure accuracy, a FIS of ANFIS model with membership functions, five output responses, and five input responses must be constructed. The proposed architecture of the ANFIS model comprises five input parameters and one output value, as displayed in Fig. 1. Several parameters must be verified one at a time. For every input variable, including particle size (*X*_1_), methanol concentration (*X*_2_), ultrasound exposure time (*X*_3_), temperature (*X*_4_), and ultrasound intensity (*X*_5_), there are three fuzzy sets: low, medium, and high. Similarly, experimental results on predicted output responses were TPC (670 mg gallic acid equivalents (GAE)/g), TFC (451 mg rutin equivalents (RE)/g), DPPH*sc (81.2%), ABTS*sc (77.4%), and FRAP (71.6 μg mol (Fe (II))/g) were defined in five fuzzy sets namely very low, low, medium, high and very high. Experiment data and human observation data were utilized to construct the fuzzy rule. The fuzzy inference system had a total number of fuzzy rules 324 and a number of network nodes 664 (Number of input response 5, output response 1 (at a time), and the type of membership function is Gaussian) presented. The predicted values of the responses were utilized to improve the fuzzy rules through RSM.

### Machine learning algorithm

**Figure 4:**
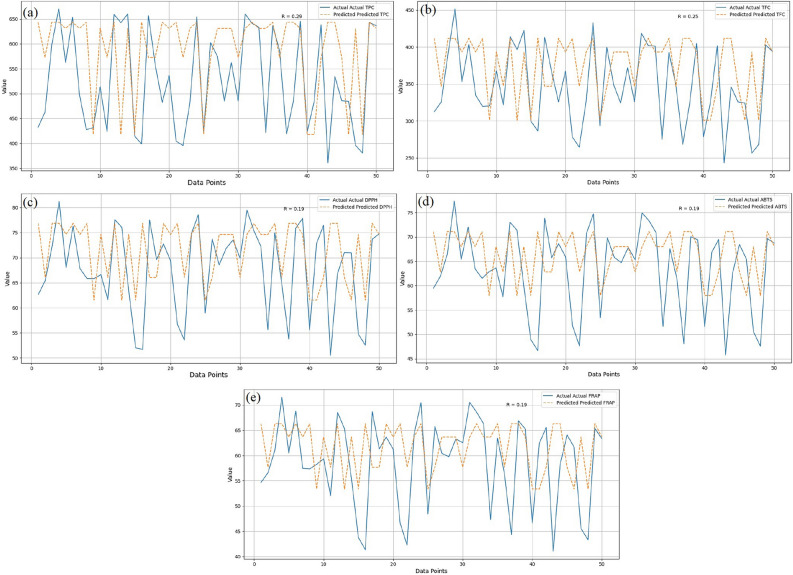
Machine learning algorithm validated the experimental and predicted values.

The inputs are the characteristics of the experimental parameters (*X*_1_, *X*_2_, *X*_3_, *X*_4_, and *X*_5_), and the output responses are y_1_, y_2_, y_3_, y_4_, and y_5_. The dataset contains the five goal columns. Thus, the five random forest regressor models were constructed by maintaining the input data constant and changing the output response for each model. This experiment’s estimators are set to 100. The R error value is used to evaluate the models after they have been fitted to the training set of data. Subsequently, the models predict the input data (*X*_1_: 0.1554 mm particle size, *X*_2_: 65% methanol concentration, *X*_3_: 23 min, *X*_4_: 40 °C, and *X*_5_: 70 W cm^−2^ ultrasound intensity). Total polyphenolics (643.53 mg GAE/g), total flavonoids (411.64 mg RE/g), %DPPH*sc (76.84%), %ABTS*sc (71.12%), and FRAP (66.30 μg mol (Fe (II))/g) were all expected to have the desired output responses based on the experimental results. Figure 4a–e are created for each of the five models to illustrate the error variance between the predicted and actual values.

### Verification of the model

**Figure 5:**
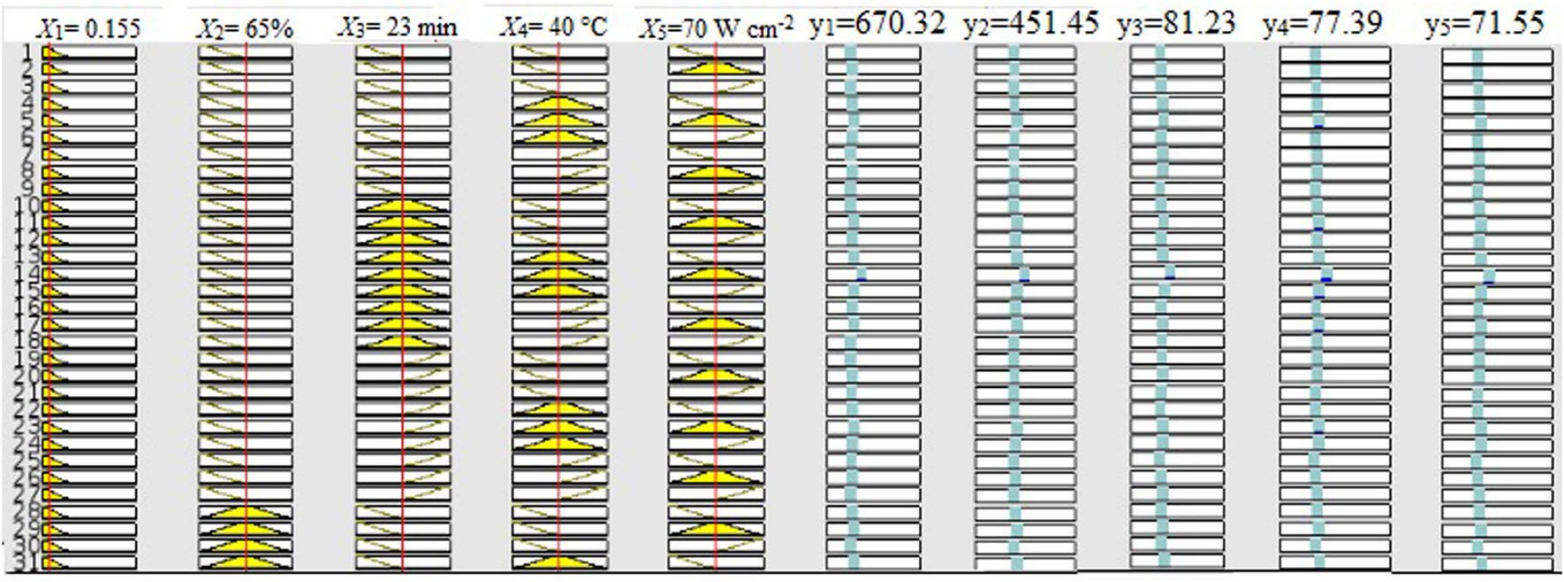
ANFIS rule viewer for the effect of extraction parameters on responses for extraction of TPC, TFC and antioxidants from grape seeds extract.

The obtained optimized extraction condition based on the CCD of RSM was confirmed with verification experiments for maximum yield of bioactive ingredients from grapes seeds extract. The significantly influenced parameters’ values slightly changed, and verification experiments were performed individually. The obtained verification experimental results feed into the Design Espert software and analyse the verification experimental results and their predicted output responses based on the yield of TPC, TFC, and antioxidant scavenging potentials (%DPPH*sc, %ABTS*sc and FRAP) from grapes seeds. ANFIS and the machine learning algorithm used the same data for further verification. The verification experimental results exhibited that the particle size, methanol concentration, and temperature significantly affected the highest yield of bioactive ingredients from grapes seeds. Table 4 displays the results of verification experiments conducted under optimized conditions and with minor modifications based on values of extraction parameters. Based on the verification experiment, 0.155 mm particle size of grapes seeds powder, 62.5% of methanol, in 23 min of ultrasonic waves exposure time, at 40 °C with 70 W cm^−2^ ultrasonic intensity, under this condition while the experimental values of TPC, TFC, and antioxidant scavenging potentials were 672. 45 mg GAE/g, 454.65 mg RE/g, 81.89%, 77.85%, and 71.52 μg mol (Fe (II))/g), respectively. Further, the predicted values from RSM models are TPC, TFC, %DPPH*sc, %ABTS*sc and FRAP were 772. 64 mg GAE/g, 469.42 mg RE/g, 82.22%, 76.72%, and 71.52 μg mol (Fe (II))/g), respectively. By changing the extraction parameter (input) values, the value of the responses (output) was observed using a rule viewer plot (Fig. 5). The rule viewer is a compressed toolbox with built-in neural weight optimization and fuzzification techniques. Implementation experiments and comparing the outcomes with the model’s predicted value allowed for additional cross-validation of the model. In the grape seeds extract, the predicted responses obtained through the ANFIS model were TPC, TFC, and antioxidant scavenging potentials (%DPPH*sc, %ABTS*sc, and FRAP) were 632 mg GAE/g, 426 mg RE/g, 76.5%, 72.8%, and 67.3 μg mol (Fe (II))/g), respectively. At the same time, the machine learning algorithm model predicted the responses, the values for TPC, TFC, and antioxidant scavenging potentials (%DPPH*sc, %ABTS*sc, and FRAP) were 669.69 mg GAE/g, 455.11 mg RE/g, 81.18%, 76.93%, and 71.14 μg mol (Fe (II))/g), respectively. According to the findings, RSM, ANFIS modelling, machine learning algorithm predictions, and the experimentally obtained values and regression analyses fit well.

**Table 4:** Central composite design (CCD) with experimental responses and predicted responses.

S.No	Parameters					Experimental value*					RSM prediction					ANFIS prediction					Machine learning algorithm prediction				
	X1	X2	X3	X4	X5	y1	y2	y3	y4	y5	y1	y2	y3	y4	y5	y1	y2	y3	y4	y5	y1	y2	y3	y4	y5
1	0.25	65	23	40	70	667.67	445.65	78.95	75.65	70.04	695.15	441.62	82.41	77.2256	72.3434	717	471	85.6	81.3	75.7	666.87	449.63	79.57	75.66	70.28
2	0.155	62.5	23	40	70	672.45	454.65	81.89	77.85	71.52	752.64	469.42	82.22	76.72	71.52	632	426	76.5	72.8	67.3	669.70	455.11	81.18	76.93	71.14
3	0.155	67.5	23	40	70	670.13	448.76	79.05	76.45	70.43	732.76	467.24	83.24	78.544	73.5081	634	427	76.8	73.3	67.8	668.34	451.40	79.52	76.10	70.49
4	0.155	65	23	40	67.5	658.65	463.34	80.34	73.78	70.37	751.71	472.56	82.26	77.4991	72.2171	635	427	76.7	73.1	67.6	662.44	458.40	80.11	74.71	70.42
5	0.155	65	23	40	72.5	659.67	462.34	81.32	74.71	70.24	735.34	465.39	83.61	78.0947	73.176	632	426	76.7	73	67.5	663.29	458.12	80.85	75.40	70.39

*All the experiments repeated three times.

### GC–MS analysis

**Figure 6:**
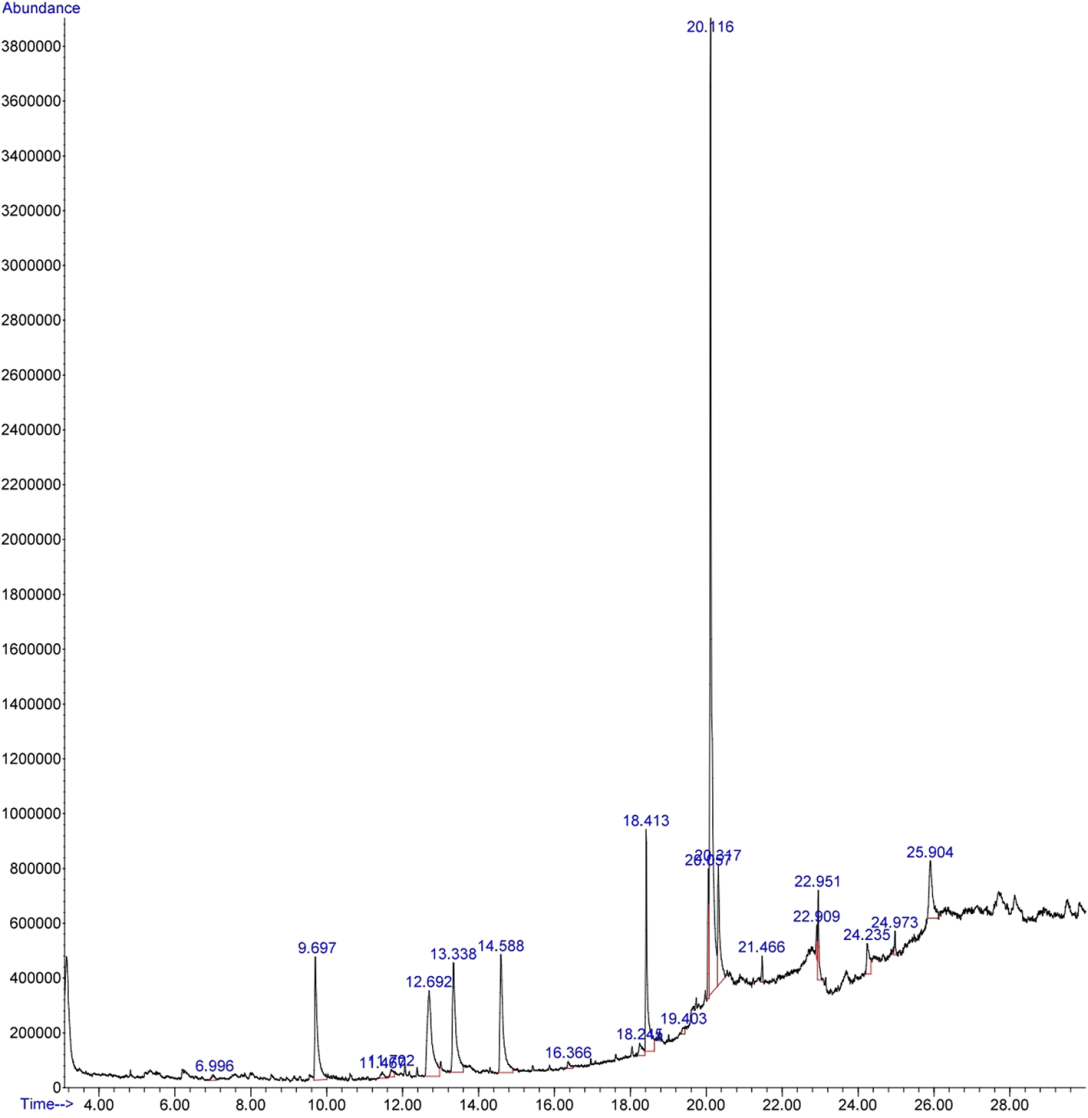
GC–MS spectra of optimally optimized extract of grape seeds. List of bioactive phytocompounds presence in the optimally obtained extract.

A total of 20 peaks were observed from optimally obtained grape seed extract of the GC–MS chromatogram (Fig. 6) by comparing the peak retention time, peak area (%), height (%), and mass spectral fragmentation patterns to those of the well-known compounds listed in the National Institute of Standards and Technology (NIST) library. Among the 20 peaks, 12 bioactive compounds (based on the active nucleus of the structure) were identified. Table 5 shows the identified bioactive compounds and their molecular formula, with molecular mass. The bioactive compounds present in the optimized extract of grapes seeds were found to be 3-Hexenoic acid, 5-Hydroxymethylfurfural, 2-Amino-5,6-dimethyl-3H-pyrimidine, Spiro[4.4]nonane-1,6-dione, 8-Methylnonanoic acid, 3,4-Altrosan, 1,5-Anhydro-d-mannitol, 9-Eicosenoic acid, *cis*-Vaccenic acid, 1,37-Octatriacontadiene, 1,3-Benzenedicarboxylic acid 2-Methyl-7-phenylindole.

**Table 5:** Analysed bioactive ingredients from optimized extract of graph seeds through GC**–**MS chromatogram*.*

Retention time (min)	% Area of peak	Compound identified	Molecular formula	Molecular weight (g/mol)
6.996	0.36	3-Hexenoic acid	C_6_H_10_O_2_	114.14
9.697	7.14	5-Hydroxymethylfurfural	C_6_H_6_O_3_	126.11
11.467	0.43	2-Amino-5,6-dimethyl-3H-pyrimidine	C_6_H_9_N_3_O	139.16
11.702	0.52	Spiro[4.4]nonane-1,6-dione	C_9_H_12_O_2_	152.19
12.692	9.22	8-Methylnonanoic acid	C_10_H_20_O_2_	172.26
13.338	8.22	3,4-Altrosan	C_6_H_10_O_5_	162.14
14.588	8.29	1,5-Anhydro-d-mannitol	C_6_H_12_O_5_	164.16
16.366	0.38	9-Eicosenoic acid	C_20_H_38_O_2_	310.5
18.245	1.00	*cis*-Vaccenic acid	C_18_H_34_O_2_	282.5
22.909	0.68	1,37-Octatriacontadiene	C_38_H_74_	531
24.235	1.91	1,3-Benzenedicarboxylic acid	C_8_H_6_O_4_	166.13
24.973	0.56	2-Methyl-7-phenylindole	C_15_H_13_N	207.27

### LC–MS analysis

Liquid chromatography coupled with mass spectroscopy is one of the significant tools for the structural identification of small molecules in the grape seed extract. Both positive and negative modes of LC–MS chromatogram of optimally obtained grape seed extract were presented in Supplementary Fig. S1. Further, the chromatogram (Supplementary Fig. S2) both positive and negative modes showed many peaks at different retention times and specified the presence of four active compounds, in the positive three active compounds identified, namely, catechin (retention time: 6.242 min; molecular formula: C_15_H_14_O_6_; mass 290.27 g/m), (−)-epicatechin (retention time: 5.262 min; molecular formula: C_15_H_14_O_6_; mass 290.27 g/m), Fisetinidol (retention time: 4.983 min; molecular formula: C_15_H1_4_O_5_; mass 228.24 g/m). and *trans*-resveratrol (retention time: 6.899 min; molecular formula: C_14_H_12_O_3_; mass 274.27 g/m), and in the positive mode one compound identified (−)-epicatechin-3-*O*-gallate (retention time: 9.284 min; molecular formula: C_22_H_18_O_10_; mass 442.4 g/m).

## Conclusion

The most effective extraction parameters for a high yield of bioactive components from powdered grape seeds were optimized using a statistical analysis technique based on the CCD of RSM. The experiments were performed per the design of a well-fitted model for extracting the highest yield of TPC, TFC and free radical scavenging potentials (%DPPH*sc, %ABTS*sc, and FRAP) from graph seeds powder. The optimized parameters are further verified through robust ANFIS and machine learning algorithm techniques. The obtained results demonstrated that the independent variables of linear term (particle size (*X*_1_) and temperature (*X*_4_), interaction terms (*X*_2_*X*_4_, *X*_3_*X*_5_, and *X*_4_*X*_5_), and quadratic terms *X*_2_^2^, *X*_3_^2^, and *X*_5_^2^) potentially contributed to the maximum yield of bioactive ingredients from grape seeds powder. Combining all five parameters significantly enhances the yield of bioactive ingredients. The observed ideal experimental values were verified and found to fit both the observed and anticipated values using second-order polynomial equations. The design’s high R^2^ value (>> 0.8) confirmed the model’s reliability. The optimal condition was observed at 0.155 mm particle size (*X*_1_), 65% methanol concentration (*X*_2_), 23 min ultrasound exposure time (*X*_3_), temperature (*X*_4_) at 40 °C, and ultrasound intensity (*X*_5_) was 70 W cm^−2^. Under this optimal condition, the highest yield of TPC, TFC, and antioxidant activities was 670.32 mg GAE/g of TPC, 451.45 mg RE/g of TFC, 81.23% DPPH*sc, 77.39% ABTS*sc and 71.55 μg mol (Fe(II))/g FRAP obtained. The optimized variable values were well matched with predicted values of RSM, ANFIS, and machine learning algorithm models. Furthermore, 12 volatile and five non-volatile natures of the bioactive compounds were recognized from the optimized extract of grape seed powder with the help of GC–MS and LC–MS spectroscopy. The optimization results from RSM coupled with ANFIS and machine learning algorithm is anticipated to help develop industrial-scale extraction procedures for the bioactive ingredients under research from grape seeds powder and related plant materials.

### Supplementary Information

Supplementary Information.

## Data Availability

All data generated or analysed during this study are included in this published article [and its Supplementary Information files].
