# Supplementary material for: Optimization of ultrasound-aided extraction of bioactive ingredients from Vitis vinifera seeds using RSM and ANFIS modeling with machine learning algorithm
Source: Sci Rep. 2024 Jan 12;14:1219. doi: 10.1038/s41598-023-49839-y (PMC10786918; doi:10.1038/s41598-023-49839-y)
Supplement: Supplementary file 1 — Supplementary Information. [file 41598_2023_49839_MOESM1_ESM.doc]

Supplementary File S1

**Optimization of ultrasound-aided extraction of bioactive ingredients from Vitis vinifera seeds using RSM and ANFIS modeling with Machine Learning algorithm**

Selvaraj Kunjiappan^1*^, Lokesh Kumar Ramasamy^2^, Suthendran Kannan^3^, Parasuraman Pavadai^4^, Panneerselvam Theivendren^5^, Ponnusamy Palanisamy^6*^

^1^Department of Biotechnology, Kalasalingam Academy of Research and Education, Krishnankoil 626126, Tamilnadu, India.

^2^School of Computer Science and Engineering, Vellore Institute of Technology, Vellore 632014, Tamilnadu, India.

^3^Department of Information Technology, Kalasalingam Academy of Research and Education, Krishnankoil 626126, Tamilnadu, India.

^4^Department of Pharmaceutical Chemistry, Faculty of Pharmacy, M.S. Ramaiah University of Applied Sciences, Bengaluru 560054, India.

^5^Department of Pharmaceutical Chemistry, Swamy Vivekanandha College of Pharmacy, Tiruchengode 637205, India

^6^School of Mechanical Engineering, Vellore Institute of Technology, Vellore 632014, Tamilnadu, India

***Address to correspondence**

Dr. Selvaraj Kunjiappan, Assistant Professor, Ph: +919994972108;

Email: [selvaraj.k@klu.ac.in](mailto:selvaraj.k@klu.ac.in)

Dr. Ponnusamy Palanisamy, Associate Professor, Ph: +919965022606;

Email: [sreepons@gmail.com](mailto:sreepons@gmail.com)

Table s1. Experimental range of coded values for central composite design (CCD)

| Independent variables (xj) | Units | Symbol | Coded variable ranges | | | | |
| --- | --- | --- | --- | --- | --- | --- | --- |
|  |  |  | -2  (Very low) | -1 (Low) | 0 (Medium) | +1 (High) | +2 (Very high) |
| Particle size | mm | *X*_1_ | 0.155 | 0.5 | 0.75 | 1 | 1.35 |
| Solvent concentration | % | *X*_2_ | 53.11 | 60 | 65 | 70 | 76.89 |
| Ultrasonic exposure Time | min | *X*_3_ | 11.11 | 18 | 23 | 28 | 34.89 |
| Temperature | °C | *X*_4_ | 28.11 | 35 | 40 | 45 | 51.89 |
| Ultrasound intensity | W cm^-2^ | *X*_5_ | 58.11 | 65 | 70 | 75 | 81.89 |


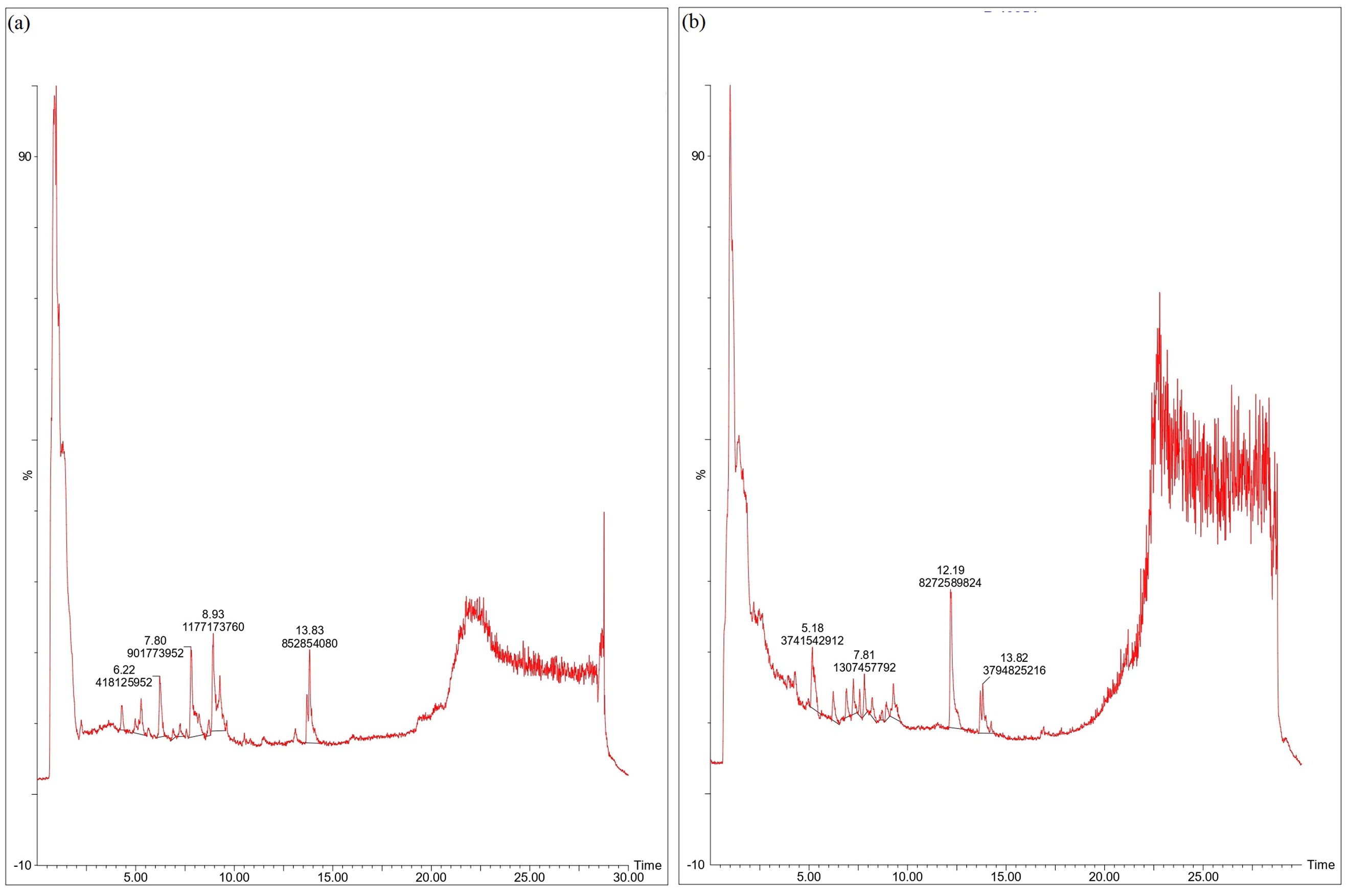


Figure s1: LC-MS chromatogram of optimized extract of grape seeds extract.


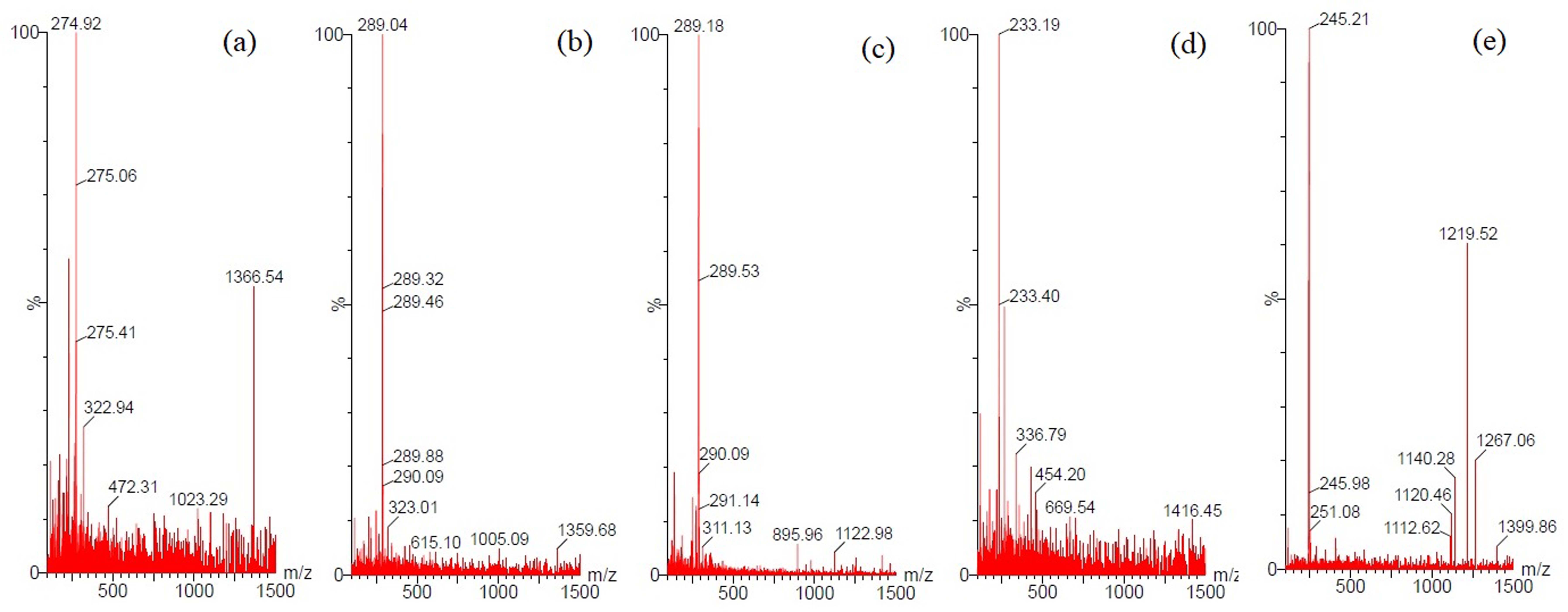


Figure s2: LC-MS chromatogram of catechin (a), (-)-epicatechin (b), (-)-epicatechin-3-O-gallate (c), Fisetinidol (d). and trans- resveratrol (e).
